# Exploring the use of social network interventions for adults with mental health difficulties: a systematic review and narrative synthesis

**DOI:** 10.1186/s12888-023-04881-y

**Published:** 2023-07-07

**Authors:** Helen Brooks, Angela Devereux-Fitzgerald, Laura Richmond, Neil Caton, Mary Gemma Cherry, Penny Bee, Karina Lovell, James Downs, Bethan Mair Edwards, Ivaylo Vassilev, Laura Bush, Anne Rogers

**Affiliations:** 1 Mental Health Research Group, Division of Nursing, Midwifery and Social Work, School of Health Sciences, Faculty of Biology, Medicine and Health, University of Manchester, Manchester Academic Health Science Centre, Manchester, M13 9PL UK; 2 Department of Clinical, Education & Health Psychology, University College London, London, UK; 3 Patient and Public Involvement Contributor, University of Manchester, Manchester, UK; 4 Department of Primary Care and Mental Health, Institute of Population Health, University of Liverpool, Liverpool, UK; 5 Linda McCartney Centre, Liverpool University Hospitals NHS Trust, Prescot St, Liverpool, UK; 6 Greater Manchester Mental Health NHS Foundation Trust, Manchester, UK; 7 Patient and Public Involvement Contributor, Cambridge, UK; 8 School of Health Sciences, University of Manchester, Manchester, UK; 9 NIHR CLAHRC Wessex, Faculty of Health Sciences, University of Southampton, Southampton, UK; 10 Public Contributor, Manchester, UK

**Keywords:** Social networks, Mental health, Implementation, Patient and public involvement, Systematic review

## Abstract

**Background:**

People with mental health difficulties often experience social isolation. The importance of interventions to enhance social networks and reduce this isolation is increasingly being recognised. However, the literature has not yet been systematically reviewed with regards to how these are best used. This narrative synthesis aimed to investigate the role of social network interventions for people with mental health difficulties and identify barriers and facilitators to effective delivery. This was undertaken with a view to understanding how social network interventions might work best in the mental health field.

**Methods:**

Systematic searches using combinations of synonyms for mental health difficulties and social network interventions were undertaken across 7 databases (MEDLINE, Embase, PsycINFO, CINAHL, Cochrane Library, Web of Science) and 2 grey literature databases (EThoS and OpenGrey) from their inception to October 2021. We included studies reporting primary qualitative and quantitative data from all study types relating to the use of social network interventions for people with mental health difficulties. The quality of included studies was assessed using the Mixed Methods Appraisal Tool. Data were extracted and synthesised narratively.

**Results:**

The review included 54 studies, reporting data from 6,249 participants. Social network interventions were generally beneficial for people with mental health difficulties but heterogeneity in intervention type, implementation and evaluation made it difficult to draw definitive conclusions. Interventions worked best when they (1) were personalised to individual needs, interests and health, (2) were delivered outside formal health services and (3) provided the opportunity to engage in authentic valued activities. Several barriers to access were identified which, without careful consideration could exacerbate existing health inequalities. Further research is required to fully understand condition-specific barriers which may limit access to, and efficacy of, interventions.

**Conclusions:**

Strategies for improving social networks for people with mental health difficulties should focus on supporting engagement with personalised and supported social activities outside of formal mental health services. To optimise access and uptake, accessibility barriers should be carefully considered within implementation contexts and equality, diversity and inclusion should be prioritised in intervention design, delivery and evaluation and in future research.

**Supplementary Information:**

The online version contains supplementary material available at 10.1186/s12888-023-04881-y.

## Background

Mental health difficulties are increasingly globally and are one of the primary drivers of disability worldwide [1, 2]. In the UK alone, 3.3 million adults in the United Kingdom (UK) were referred to mental health services between 2020 and 2021 [3]. More disability-adjusted life years are lost to mental health difficulties than to any other health condition in the UK, including cancer and heart disease, with considerable economic, societal and individual cost [4]. Adults with severe and/or enduring mental health difficulties, such as schizophrenia and bipolar disorder, face additional challenges; they are at greater risk of multiple physical health comorbidities, and have a 15-20-year shorter life expectancy than the general population [5, 6]. Optimising the effectiveness and reach of mental health support for these people is essential to ensure high-quality care whilst minimising pressures on already-stretched NHS resources.

Community engagement and social connections can support people living with mental health difficulties in the community, sometimes preventing the need for the involvement of formal health service provision and providing support for recovery post-discharge [7]. Community engagement is often used as a proxy measure of community integration which is considered a fundamental aspect of recovery from mental health difficulties [8]. Evidence suggests that both close and distal social network support are associated with community integration [9]. However, recent research suggests both individual and wider barriers to community engagement [10]. This highlights the potential value of interventions designed specifically to mitigate both the individual level barriers such as physical and psychological capabilities and social barriers which reduce access to suitable community resources [10].

Social networks^1^, social connectivity and engagement in valued activities have multiple benefits for people with severe and/or enduring mental health difficulties and associated benefits for the services and people that support them. They enhance recovery and self-management, promote engagement with community-based support and extend the availability of heterogenous support for the secondary prevention of mental health difficulties, with potential to reduce direct healthcare costs [7, 12–16]. It is theorised that formal and informal social support, interpersonal contact, and mobilisation of resources enhance individual coping strategies and functional support [17, 18], thereby providing protection from stress and improving daily self-management of mental health difficulties [19–21]. In turn, social activity can increase the size and quality of an individual’s social network [22], further sustaining and enhancing social connectivity and well-being promotion [14].

The usefulness of social networks is contingent on the availability of requisite knowledge, understanding and willingness to provide help within networks. These are not always present, available or acceptable to individuals [23]. People with mental health difficulties tend to have smaller, less diverse networks of poorer quality and configuration, and tend to rely heavily on support from family members or health professionals [24, 25]. Social network availability and configuration varies depending on the severity of mental health difficulties and availability of resource [26].

Interventions designed to improve people’s social networks by connecting them with meaningful and valued activities, people, and places, can extend access to support, thus aiding and sustaining recovery [25]. These interventions can be effective in optimising social connections for people with mental health difficulties [12]. It is important to note that social network interventions include those that strive to modify the composition or size of social networks by adding new members and those that seek to bring together existing network members to modify existing links to enhance the functional quality of a network. The former includes linking individuals to new activities or social situations where new network connections can be made [27] whilst the latter often take the form of network meetings which dependent on an individual’s personal situation bring together relevant network members (family, friends and other supporters) in order to optimise the consistency and connectedness of network support [28]. However, specific attention needs to be paid to implementation of these types of interventions because previous research in other fields suggest variability in uptake of network interventions, fluctuating capacity of organisations to deliver such interventions and organisational cultures which do not allow for sustainable implementation [29, 30].

To fully translate social network interventions into mainstream mental health services and optimise their use, we must first ascertain how effective, acceptable and feasible existing interventions are, and understand their mechanisms of effect [12]. A recent systematic review examining the effectiveness, acceptability, feasibility and cost-effectiveness of existing interventions concluded that extant literature is in its infancy, but suggested that social network interventions which connect and support people to engage in social activities may be acceptable, economically viable and effective [12]. The aim of this review is to build on these findings by providing a critical overview of how social network interventions might work best for adults with mental health difficulties. We used systematic review methods to critically answer the following questions: for people with mental health difficulties, (i) what social network interventions work best and for whom; and (ii) what are the optimal conditions for implementing social network interventions?

## Methods

Preferred Reporting Items for Systematic Reviews and Meta-Analysis (PRISMA) guidance [31] informed the methods and reporting of this systematic review and narrative synthesis, and the protocol is available from: https://www.crd.york.ac.uk/prospero/display_record.php?ID=CRD42020206490.

### Eligibility

Published journal articles or dissertations reporting primary data on the use of interventions designed specifically to improve and/or measure social network quantity or quality for people with mental health difficulties were included in this review. Review articles were excluded but reference lists of identified reviews were checked for potentially relevant articles. Only studies with a sample mean age ≥ 18 years and a minimum of 75% of participants with primary diagnosis of mental health difficulties (self-report or physician defined) were included.

No restrictions were imposed in relation to language or date of publication. Non-English language articles were screened for eligibility by native speakers affiliated with the research team. Papers where the sample held a primary diagnosis of substance misuse, autism spectrum disorders, dementia, attention deficit hyperactivity disorder (ADHD), or cognitive impairment were excluded. Also excluded were dyadic interventions or individual-level interventions such as purely social skills/cognition programmes. Table 1 displays full inclusion and exclusion criteria.

**Table 1:** Inclusion/exclusion criteria

*Inclusion criteria*	*Exclusion criteria*
Peer-reviewed journal articles or dissertations.	Studies that do not report primary data
Studies reporting primary data on the implementation of interventions which are designed to improve the quantity and/or quality of social networks and/or related community-level social network properties.	Studies only available in abstract format.
Studies including adults with a primary diagnosis of mental health difficulties or self-attribution/non-medical labelling (e.g., stress or emotional distress). In mixed samples, mean age must be 18 years or over and 75% of sample must have primary diagnosis of mental health difficulties (self-report or physician defined).	Studies unavailable to the research team.
Studies which have a primary aim of improving social network quantity or quality and/or include a measure of social network quantity or quality.	Studies where primary diagnosis is substance misuse, autism, dementia, ADHD, cognitive impairment or spectrum disorders.
	Studies including participants without a primary diagnosis (or self-attribution) of mental health difficulties (or less than 75% of the sample has a primary diagnosis/self-attribution of mental health difficulties).
	Studies not related to the implementation of interventions which are designed to directly improve the quantity or quality of social networks for people with mental health difficulties (conceptualised as a whole network approach). The following were excluded: 1. Dyadic interventions – couples, individual friendship interventions (interventions which target multiple friendships can be included), family level only. 2. Individual level intervention – e.g., intervention which aims to improve individual social skills, social cognitions, confidence in social interaction, perceptions about social interaction, social interaction intentions, employment skills. 3. Pharmacological interventions
	Studies which only report on social functioning or social support without reference to the quantity or quality of social networks.

### Search strategy

The following seven databases were searched in August 2020 with searches updated in October 2021: Medline, Embase, PsycINFO, CINAHL, Cochrane Library, and Web of Science. Published reviews and literature on social network interventions informed the search strategy which was agreed with the wider authorship team and was subject to a Peer Review of Electronic Search Strategies (PRESS) review by an expert librarian [32]. Search terms were organised using the first two components of the Population, Intervention, Comparator, Outcome (PICO) framework (Population: People with a diagnosis of mental health difficulties or self-reported emotional distress and Intervention: Social network) and were intentionally broad to maximise search returns (see Additional File 1 for an example search syntax).

To reduce publication bias, grey literature sites such as EThoS and OpenGrey were also searched. We also contacted authors of possibly eligible conference abstracts for full manuscripts where these were not readily available and examined identified review articles and book chapters for relevant literature.

### Data extraction

The data management software Covidence (http://www.covidence.org) was used to aid the data selection and extraction process. After duplicates were removed, titles and abstracts of identified studies were screened independently by two reviewers, and conflicts were resolved by a third reviewer in line with the inclusion/exclusion criteria (See Table 1). Full text reviews of potential papers were undertaken by the team, with two reviewers independently screening each paper and conflicts resolved by consensus.

Standardised data extraction forms were created in Excel and used to extract data from all eligible papers by seven team members (HB, ADF, LR, NC, JD and BME), including both academic and patient and public involvement (PPI) researchers. Extracted data included social network measures (where applicable), factors of context, mechanisms and outcomes, acceptability and standard demographics. Interventions were categorised into broad groups for the purposes of analysis by one member of the research team (HB) and checked for accuracy by another (MGC). For qualitative data, both raw data quotations and author interpretations were extracted where applicable and identified as such. A second reviewer from those outlined above (HB and ADF) cross-checked 30% of extracted data from each member of the review team for accuracy.

### Quality appraisal

The Mixed Methods Appraisal Tool (MMAT) [33] was used to assess the quality of the included papers, applicable to the broad range of research arising in this review [34]. Each full-text paper was quality assessed by one reviewer in parallel with data extraction with 10% of quality appraisals cross-checked for accuracy. Disagreements were resolved through consensus.

### Data synthesis

Meta-analysis of the quantitative data was not possible due to the heterogeneity of included studies. Consequently, a narrative synthesis was undertaken following the stages outlined in the Guidance on the Conduct of Narrative Synthesis in Systematic Reviews [35]. Data were collated and textual summaries of study characteristics were produced via data extraction spreadsheets (including intervention content, study design, participants, recruitment and delivery). Qualitative data from qualitative and mixed methods studies were explored inductively using aspects of thematic synthesis: a thematic framework was developed consisting of themes which were refined, merged, split or created, as necessary, with analysis of each study [36]. Constant comparison was used to translate concepts between studies [37, 38]. Verification of findings were provided by a second researcher and verified by the wider team. The thematic framework was also applied to the quantitative data from quantitative studies and mixed-methods studies, which aided with the visual grouping of patterns across the whole data set. Quality appraisals were used to assess the robustness of the thematic analysis by removing papers of the lowest quality from each theme to consider their impact on overall presentation. No themes needed to be revised following this process so references were added back into the synthesis [35]. Data from all included studies were also grouped on aspects of context such as delivery setting and approach, diagnosis and significance of results [35]. Analytical themes were inferred from the material inherent in the descriptive findings and, together with the patterns apparent across the whole data set formed the narrative synthesis. This synthesis allowed interpretation of the concepts arising in this review beyond the primary findings of individual papers.

## Results

**Fig. 1:**
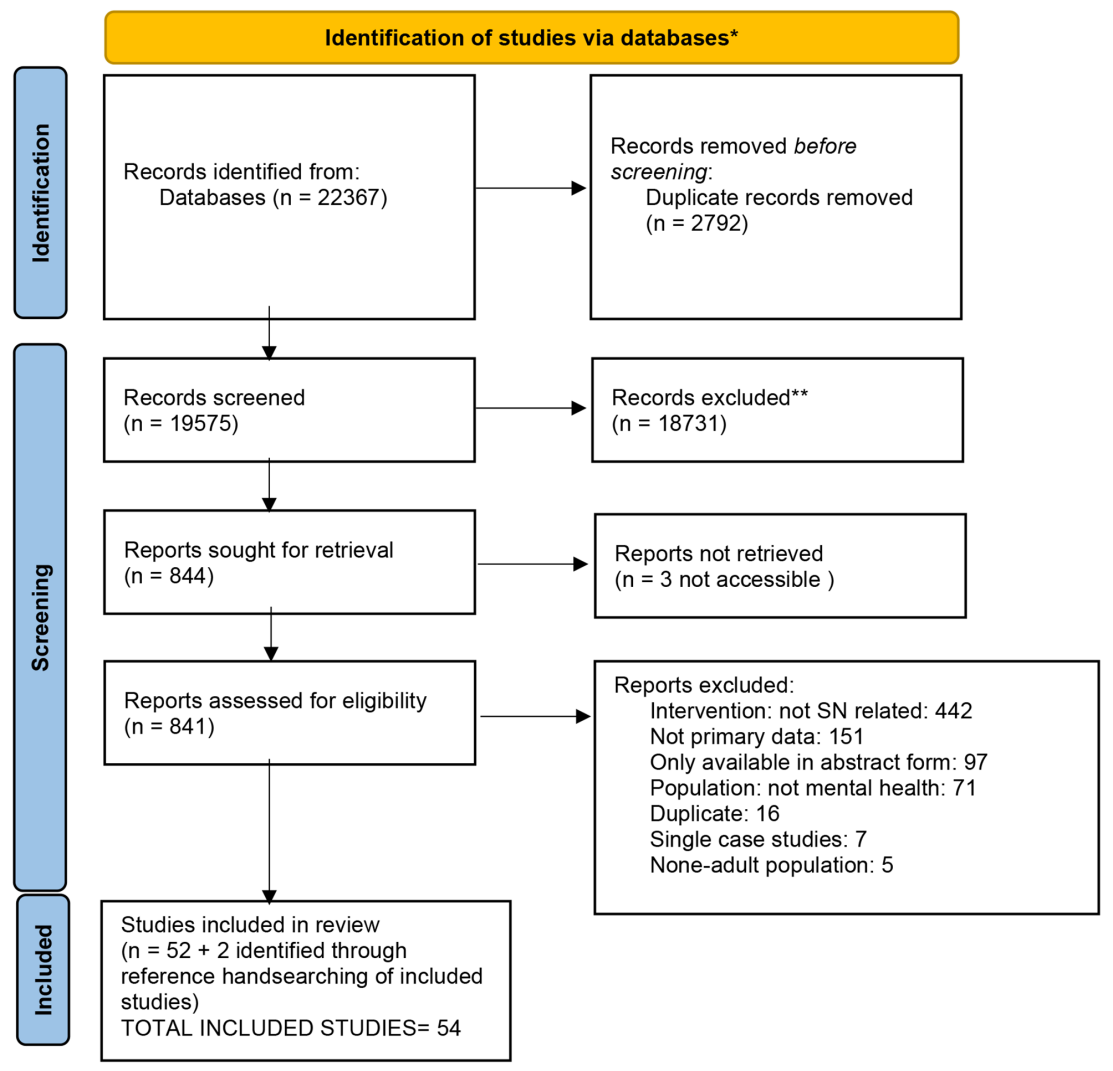
PRISMA 2020 flow diagram

As shown in Fig. 1, searches identified 22,367 potentially relevant studies, resulting in 19,575 unique citations after de-duplication. The full texts of 841 studies were reviewed for relevance, resulting in the inclusion of 54 unique papers. Of these, 17 were randomised controlled trials (RCTs), 12 were quantitative studies of other designs (‘other quantitative studies’), 13 studies were qualitative, and 12 used mixed methods. Studies were conducted in the following countries: UK (n = 25), United States of America (n = 8), Australia (n = 5), China, India, Ireland, Italy, Netherlands, Sweden and Canada (all n = 2), Denmark and Hungary (n = 1). For more information on included studies, see Additional File 2 and Additional File 3 for a completed PRISMA checklist.

There was a total of 6,249 participants recruited across the included studies with an average age of 47.42 years and broadly equivalent numbers of males and females. Most (21/54) studies recruited participants with mixed forms of mental health difficulties and emotional distress. The remaining studies included only participants with the following diagnoses or self-reported difficulties^2^:

- Psychosis and/or schizophrenia (n = 12).
- Serious and/or long-term mental health difficulties (n = 10).
- Depression (n = 4).
- Mild to moderate mental health difficulties (n = 2).
- DSM AXIS 1 disorders (e.g. anxiety disorders, such as panic disorder, social anxiety disorder, and post-traumatic stress disorder) (n = 2).
- Psychotic and affective disorders (n = 1).
- Eating disorder (n = 1).
- PTSD and depression (n = 1).

For more information on participants in included studies, see Additional File 2.

The 54 included studies reported on 51 unique interventions which were broadly categorised into five types. The most commonly reported interventions were those that supported community or social activities (25/51). These included 13 interventions that supported access to existing community resources and activities, 3 football interventions, 5 horticulture or nature-based interventions, 3 arts-based intervention and one which involved closed group social activities. The second most included type of intervention (13/51) was intensive or enhanced community treatment. These were mostly assertive community treatment (n = 3), case management approaches (n = 3) and specialised community treatment teams (n = 2). There was also one reported example of each of the following: day centre, community club, social recreation team, occupational therapy and rehabilitation specialised services. There were 7 peer support group interventions within included studies and 3 one-to-one interventions (behavioural activation, cognitive behavioural therapy and peer-led recovery). Three interventions were classified as other and these included 2 action research approaches and an enhanced sheltered accommodation project. Please see Table S1 in the additional files for additional detail on included interventions.

23/54 intervention activities were additional to statutory provision and delivered externally to statutory services, 9 were additional but were delivered within health services, 1 was a combination of both internally and externally delivered activities and 21 were designed to enhance existing provision.

There was minimal description of formal patient and public involvement (PPI) in the included studies, with notable few exceptions (n = 10; [39–48]). PPI activities included participatory approaches such as Photovoice [39], co-production activities [47], peer researchers/facilitators [40–42, 44] and inclusion of advisory groups or public advisors [43, 45, 46, 48].

### Quality assessment

Details of the quality assessment of included studies is found in Additional File 2 which includes assessments for each type of study. Quality assessments and main methodological weaknesses for each type of study are summarised in Table 2.

**Table 2:** Summary of quality assessments

Type of study	Average number of quality criteria met (out of 5)	Main methodological weaknesses.
Randomised controlled trials	3 (ranging from 0–5)	• Lack of detail on whether participants adhered to the assigned intervention. • Lack of blinding of outcomes assessors or lack of detail about blinding of outcome assessors. • Lack of detail on randomisation.
Other quantitative studies	3 (ranging from 1–4)	• Lack of detail on whether the intervention was delivered as intended. • Lack of detail on how confounders were accounted for. • Lack of detail on the representativeness of the participants.
Qualitative studies	5 (ranging from 3–5)	• Coherence between qualitative data sources, collection, analysis and interpretation
Mixed methods studies	2 (ranging from 0–5)	• Lack of detail on how divergences and inconsistencies between quantitative and qualitative data were assessed. • Lack of adherence or lack of detail about adherence to the quality criteria of each tradition. • Lack of detail on the rationale for the use of a mixed methods approach.

### Review question 1: what type of social network interventions work best and for whom?

Of the 17 RCTs, 12 other quantitative and 12 mixed-methods studies included, 9 RCTs [49–57], 3 other quantitative studies [58–60] and 1 mixed-methods study [61] used included a formal quantitative measure of social network size or quality. Of these, 7 provided evidence of statistically significant improvements to social networks post intervention [49, 51, 53, 55, 56, 58, 59] and others demonstrated improvements which favoured the intervention group but did not reach statistical significance [52, 54, 62]. Just over half (5/9) of the RCTs examined the effectiveness of interventions using aspects of supported socialisation [53, 54, 56, 59, 62], highlighting the potential value of these types of interventions for people with mental health difficulties. The follow up periods for RCTs ranged from 3 months to 24 months and effect sizes were generally small to moderate when compared to usual care – ranging from 0.39 to 0.65 [12].

A range of statistically significant improvements in other outcomes following intervention were reported across the included studies. These included mental health symptomatology [44, 51, 55, 63–67], general health [68], social anxiety [69], social support, social capital and satisfaction with aspects social relationships [43, 49, 50, 70], distress [50], general and social functioning/engagement [43, 44, 54, 63–65, 71–74], occupational functioning [75], structured activity levels [76], loneliness [43, 54, 64, 69], relatedness and social inclusion [42, 44], sense of belonging [69], self-esteem [43], quality of life [43, 72], wellbeing [42, 64, 77], treatment adherence [72], service use [44, 52] and satisfaction with care [52, 72]. Of these 24 studies, 20 provided information on follow-up periods which were on average 9 months ranging from 2 to 18 months. See Additional File 2.

It was not possible from included studies to draw definitive conclusions about the groups of people for whom these interventions work best due to the heterogeneity of participants in included studies. However, 8 of the 28 studies that demonstrated significant improvements in outcomes evaluated the effectiveness of interventions for people with schizophrenia and/or psychosis. Significant improvements at follow-up across studies were also associated with:

- Being female [57, 58, 63].
- Being married [43, 58].
- Living with a spouse or partner [43].
- Completing A-levels [57].
- Fewer negative symptoms [57].
- Larger network at baseline [57].
- Better baseline functioning [63].
- Greater distress from positive symptoms [51].
- Longer duration of illness [51].
- People who demonstrated improvement in other outcomes [56].
- Undertaking more social activities [51, 65].
- Having a better clinical prognosis [56].

### What are the optimal conditions for the implementation of social network interventions?

Synthesis of data from qualitative and mixed-methods studies identified a range of barriers and facilitators to implementing social network interventions which are presented in Table 3 (Individual level barriers and facilitators) and Table 4 (Provider/agender level barriers and facilitators). Overarching themes identified during the narrative synthesis relating to optimal conditions for implementation and how interventions are thought to bring about changes in outcomes are presented below with supporting quotes presented in Table 5.

**Table 3:** Identified barriers and facilitators in the implementation of social network interventions at the individual or service user level

Barriers	Facilitators
***Socioeconomic***	***Socioeconomic***
• Lack of personal resources (Felton et al., 2009; Chowdhary, 2016) • Lack of transport (Felton et al., 2009; Chowdhary, 2016; Margrove et al., 2013) • Living in low socioeconomic areas (Hassan et al., 2020) • Financial burden of prioritising living costs (Mathias, 2019)	
***Social Support***	***Social Support***
• Lack of someone to attend with (Sheridan, 2018) • Lack of familial support (Mathias, 2019)	
***Psychological***	***Psychological***
• Social anxiety (Lund, 2019; Hanly et al., 2020; Howarth et al., 2018; Margrove, 2013) • Anxiety about end of programme activities (Darongkamas, 2011) • Previous negative experiences (Lund, 2019; Sexton, 1992) • No previous experience of group activities (Lund, 2019) • Reduced social skills (Snethen et al., 2012) • Lack of readiness for change/ engagement (Kaltman, 2016) • Amotivation (Snethen et al., 2012; Kaltman, 2016) • Stressful life events during intervention period (Van de Venter, 2014)	
***Health-Related***	***Health-Related***
• Poor physical health (Hanlon, 2019; Chowdhary, 2016) • Fatigue (Mathias, 2019) • Severity of mental health at baseline (O’Connell, 2020; Aggar, 2021)	
***Time***	***Time***
• Competing commitments (e.g. work, caring) (Sheridan, 2018; Hanly et al., 2020; Chowdhary, 2016; Margrove et al., 2013)	
***Cultural***	
• Familial expectations (Mathias, 2019) • Social hierarchy (Mathias, 2019) • Language barriers (Van de Venter, 2014) • Literacy barriers (Chowdhary, 2016)	

**Table 4:** Identified barriers and facilitators to social network interventions at the provider and agency-based level

Barriers	Facilitators
***Socioeconomic*** • Lack of funding for programme/staff/training (Bertotti, 2013) • Lack of perceived safety in local area (Sheridan, 2018)	***Socioeconomic*** • Use of existing, acceptable community resources such as allotment groups – widely available and inexpensive (Fieldhouse, 2003) • Providing financial support (small social stipend) (Sheridan, 2015; 2018) • An all-encompassing hub, which facilitated ease of access to many meaningful avenues of support and other resources (e.g. computers, library, social spaces/activities, advice, cafe) without additional financial cost. (Hassan et al., 2020)
***Social Support***	***Social Support*** • Provide someone to support engagement with activities in the early stages (Snethen, 2012; Sheridan, 2015; Rivera, 2007)
***Psychological*** • Stigma/prejudice and lack of understanding of mental health problems within intervention settings (Mathias, 2019; Lund, 2019) • Anxiety about end of programme activities (Darongkamas, 2011)	***Psychological*** • Flexible approach to opportunities provided (O’Brien, 2011) • Allow people to go at own page during activities (O’Brien, 2011) • Use of humour in delivery (Lund, 2019) • Provide someone to support engagement with community activities (Sheridan, 2018) • Individual sessions with facilitators to promote engagement (Kaltman, 2016)
***Health-Related***	***Health-Related*** • Flexible approach to opportunities provided (O’Brien, 2011) • Access barriers tackled by changing delivery/format (home visits and home work rather than attending health care facility or via telephone) (Chowdhary, 2016)
***Organisational*** • Limited scope of programme (Hanlon, 2019; • Staff resistance to programme or research methods (Hacking, 2008) • Lack of infrastructure/funding (Bertotti et al., 2018; (Webber et al., 2021) • Lack of training (Chowdhary 2016; Hanly, 2020; Bradshaw, 1998)	***Organisational*** • Safe, non-judgemental space, everybody equal, no barriers (Abotsie, 2021) • Environment that expects and structures social interactions without pressure ’ (Hacking, 2008) • Structured support for real world activities (Rivera, 2007)
***Time*** • Lack of time for staff to implement programme fully (Webber et al., 2021; • Sessions/programme not felt to be long enough for benefit (Kaltman, 2016); • Study too short to show true results (Kaltman, 2016; Rivera, 2007)	***Time*** • Flexible approach to opportunities provided (O’Brien, 2011)
***Cultural*** • Inappropriate Programme content (Mathias, 2109) • For research, lack of ethnic diversity impacted results (Van de Venter, 2014)	***Cultural*** • Literacy barriers addressed with use of icons, visual aids, staff training on literacy issues, and familiar, accessible language (Chowdhary, 2016)
***Gender*** • Lack of gender inclusion both to enter groups and within groups (Friedrich, 2018; Van de Venter, 2014 • For research imbalance of gender impacted results (Van de Venter, 2014)	***Gender*** • None identified.

**Table 5:** Supporting quotes from included studies

	Supporting quotes	Paper reference
**Bridging the gap – the fundamental role of facilitation**	*You can ask all kinds of stuff and they* [the facilitators] *were quite nice about it. They were really nice and encouraging to me. And I enjoyed it... And you know they gave us the um, binder and every week they give us different pages for the binder.*	Suto, 2020.
	*She* [the facilitator] *spoke to me about all my problems and how I was getting on. All very informal and I can cope with that, but I couldn’t cope with talking to a doctor looking at the time all the time*	Hassan, 2020.
	*She was just absolutely wonderful…she was just right…I told her what had happened and that seemed to get it out of my head a bit. All these years it’s just been in my head*	Bertotti, 2018.
	*When you’re actually going tae the doctor because you’re no’ functioning properly day-tae-day, an’ somebody takes a’ the bits that you struggle with, they’re a lifesaver. It’s better than any beta- blocker.*	Hanlon, 2019.
	*t was like she’d come down the mountain a little and throw me a rope, and it would pull me up a little bit every time… then the next time she didn’t have to come as far down the mountain…It was almost like we were getting to a point of meeting in the middle. But the middle wasn’t actually the middle of the mountain, it was closer to her top than to my bottom.*	Hanly, 2020.
	*I think [she was] concerned about my mental health, and my physical health, and my future. How so? I can tell by the way [she] treat[s] me. I think [the facilitaotr] has shown me more concern than most types all the years I’ve been here.*	Snethen, 2012.
**My voice, my choice, my pace – the need for flexibility and valuing individual differences**	*At the beginning you explained everything well and you made me feel that, in a group meeting, it would be my choice if I wanted to share something or not, and that made me feel confident.*	Kaltman, 2016.
	*I have come here a few times just to be by myself, to a safe space which is nice because obviously I can come up and get a cup of coffee if I want and sit down and they respect the fact that you want to be on your own*	Hassan, 2020.
	*I have social phobias and agoraphobia… but I decided to continue [with BEL] because I thought it helped me anyway…I have a hard time to drive by myself… the first time I was like, ugh…but it got better and better.*	Lund, 2019.
	*You can come here for say 10 min, 20 min, half an hour and just those few minutes or second or that bit of time you spend with somebody here who’s nice to you can make you feel a bit better but you are in charge of what you are doing. I think it’s really, really important and just that little bit of control can make you feel on top of the world, you can go away thinking I did something really good today.*	Hassan, 2020.
	*You can have your own creation as far as what you grow, and your choices of what you want to grow and what you want to eat and you can experiment with certain things.*	Suto, 2021.
	*At the beginning you explained everything well and you made me feel that, in a group meeting, it would be my choice if I wanted to share something or not, and that made me feel confident*	Kaltman, 2006.
***Social building blocks – rebuilding or acquiring social resources and skills and making connections with others***	*My confidence is ecstatic. The more I work, the more my confidence grows.*	Howarth, 2018.
	*Activities means that you’re not locked up, you know? When the door is out there and you can’t get out, you can’t do nothing. That’s a reminder that I can get out and do stuff, not locked behind the door*	Snethen, 2012.
	*I have always loved reading. In the past, I used to read and now I want to start reading books again to feel good…that is what I told my husband and he said, “Do you want to read? You are changing!”*	Kaltman, 2016.
	*I wasn’t one for socialising before – I used to make excuses to stay in, but now I make the effort to go out’. P1 said: ‘It’s helped me get back in contact with friends from the past… It’s encouraged me to meet people.*	Darongkamas, 2011.
	*I have thought more about things I used to do before I became ill.* *I feel more able to go out on my own.*	Bradshaw, 1998.
**The importance of a positive and safe space to support community integration**	*No judgment is passed on your capabilities or your mental well-being. I attend as many sessions as I can.*	Abotsie, 2021.
	*To be in a group…[I thought it would be] like the worst thing I know. So that I was able to just spontaneously begin talking and discussing with others…. and afterwards think, my God, what am I doing? (laughter) So it was a good group.*	Lund, 2019.
	*It’s a lovely building, big room, lots of light /* *It’s not claustrophobic /* *The room is just perfect to work in /* *You felt you could move around, it was loose and fluid from the beginning /* *A very relaxing atmosphere.*	Margrove, 2013
	*You’ve got, you’ve got to be able to… You’ve got to feel relaxed in your environment and here I think most of us are relaxed from my view anyway, the ones that I talk to…I mean we enjoy each other’s company which is a good thing.*	Friedrich, 2018.
	*But, this group, it was probably the world’s best group I have been in, because we could discuss everything []. You get to know one another; it’s like reading someone’s diary. After only three weeks, you didn’t know each other’s shoe size, but you knew about their childhood… To be in a group where people are kind to each other, that heals the worst ulcer. You feel that no one is going to say something mean… just the opposite, be positive.*	Lund, 2019.
	*“It’s just such a safe place because, even if I am not in a good mood, I get out and at least go to the Life Rooms*	Hassan, 2020.
**The need for available, accessible and sustainable activities**	*How can they act together when they are all working so hard? They are so busy at the brick kiln: we wake them at midnight to go to make bricks and they return 11am and are so tired that they just want to sleep. Who will they speak with, they don’t even want to eat, just sleep.*	Mathias, 2019
	*“[in other services] you would have to fill in loads of forms or you would have to apply online or you would have to get some type of funding, but since I have been here, I have done loads of stuff and I have never been asked for a penny.*	Hassan, 2020.
	*I very much appreciate the money [stipend to support acces]. I was able to socialise with cups of coffee meeting people and chatting.*	Sheridan, 2018.
	*One day one of my friends also came with me to the group. The next day he told me that when he went back (home) after the group, people teased him and called him names. They said that the boys in the group were not considered to be good boys and due to this problem, he couldn’t continue in the group.*	Mathias, 2019.
	*I’m worried about the club folding, it makes me anxious.*	Darongkamas, 2011.

#### Bridging the gap – the fundamental role of facilitation

Facilitators played a central role in the successful implementation of social network interventions for all types of mental health difficulties. A facilitator could support the initiation of social activity through personalised discussions about activity options and going along to activities with an individual until they had developed sufficient skills, knowledge and confidence to undertake activities on their own [39, 40, 42, 46, 48, 62, 67, 78–82]. To do this well, facilitators needed to have sufficient local knowledge, empathy and engagement skills [83, 84]. The development of interpersonal trust and provision of suitable options for an individual to consider were considered key to successful facilitation [41, 84]. Other requirements included being non-judgemental, approachable, friendly, and having a basic understanding of mental health difficulties [41, 42, 48, 62, 66, 67, 74, 82, 85]. A mutual understanding and respect of roles and boundaries was also crucial to successful facilitation [79].

Participants described how facilitators needed to maintain a delicate balance between providing support to engage with new social activities and promoting independence to ensure future sustainability [39, 46, 83]. Facilitators could support the uptake of social activities by providing structured programmes with sufficient flexibility to overcome individual barriers to accessing local activities [40]. Studies also highlighted the need for adequate training and supervision for facilitators in advance of programmes starting and for consideration to be given to the end of interventions when contact with the facilitator ended. Sufficient facilitator relationships coming to an end and for consideration of how benefits would be sustained once the programme ended [67, 79, 81].

Whilst there wasequivalence in the quantitative effectiveness data in relation to peer versus non-peer facilitators [12], qualitative data identified particular strengths of peer facilitators in relation to having shared experiences and having opportunities to model behaviours. Peer facilitators were seen to provide hope for the future as an example of someone who had recovered from a mental health difficulty, and also to reduce the imbalance of power between facilitator and service user, which improved their relationship [41, 79].

Social network interventions could benefit facilitators and host organisations by increasing knowledge about, and access to community infrastructure which provided s ongoing support to service users. Additionally, professionals were able to develop more in-depth understanding of individual service users during such interventions, which could improve understanding about individual triggers of distress and relapse [67, 79].

#### My voice, my choice, my pace – the need for flexibility and valuing individual differences

Social network interventions worked best when service users felt they could choose activities within interventions which mirrored their own interests [46, 48, 67]. This improved uptake and engagement with social activities particularly when users felt that their voices were being heard and their choices considered [39, 40, 83, 84].

Acknowledgement of individual differences and allowing people to be who they are whilst providing gentle encouragement appeared to increase engagement with valued activities [46, 62, 79, 86]. Participants, particularly those with serious and/or enduring mental health difficulties, experienced increased motivation for, and enjoyment of, self-selected activities [79, 82, 86]. Participants described such activities as evoking a sense of fun and spontaneity which helped them to be playful and self-expressive [77, 79, 87] as well as to laugh and be adventurous [41]. Engagement with valued activities was seen as empowering and participants expressed an increased desire for future engagement, feeling as though they were seen as a person rather than an ‘illness’ [42, 62, 88]. Participation was optimised if space was provided to allow people to try different activities and ascertain what was most enjoyable for them. This allowed people to become familiar with, and embedded into, intervention locations [48, 66, 74].

Participants described the impact that their mental health and other external circumstances could have on their ability or readiness to engage with social network interventions. Studies recommended flexibility in implementation to mitigate against this [67, 77, 78, 80, 82]. Interventions worked best when participants felt that they could be honest in relation to their own boundaries/capabilities [80] and when they could be left alone when they needed to be [46]. This flexibility and acceptance of individual situations meant people felt their own needs, choices and health were being adequately considered, which allowed them to push themselves further than they might have thought possible [46, 80]. It also appeared to contribute to a sense of agency and control over their own participation which was deemed important for successful engagement with social network interventions [46, 82]. Not having individual needs met through a lack of flexibility could result in withdrawal from intervention activities [81].

Another key feature of successful social network interventions was allowing participants to progress at their own pace, one that was manageable given their individual circumstances [62, 79, 82, 85, 89]. Any pressure to move faster than this or at another’s pace was viewed as a potential barrier to these types of interventions. One study with people with serious mental health difficulties found that those who engaged with social activities independently were more consistent and committed in their engagement, and this was attributed to the ability to go at their own pace [78].

Similarly, social network interventions should not be seen as a quick fix or panacea for people with mental health difficulties. What is experienced as valuable and beneficial for one person is likely to be different for another and individual preferences may change over time. These types of interventions need to be personalised to individuals to ensure they meet people’s needs and that expectations for engagement are realistic for the individual [46, 67, 79]. It was recognised, within included studies, that not everyone would be able to engage with social network interventions, and this should be factored in from the outset and a flexible approach undertaken [79, 80]. Flexibility in delivery also incorporated the ability to include the wider family, friends and other supporters in intervention activities where appropriate and desired [46].

#### Social building blocks – rebuilding or acquiring social resources and skills and making connections with others

Social network interventions were considered to work best when they enabled individuals to build on existing or develop new skills whilst also being supported to make connections with others [45, 83, 86]. This applied at an individual level (self-esteem, self-efficacy, resilience, social skills, self-management) and social network level (quantity and quality of new and existing social networks) [39, 41, 42, 45, 46, 48, 77, 78, 81–83, 87–89]. Individual-level improvements were considered necessary in order to realise benefits from social network interventions [84]. Such benefits could be conferred formally through didactic sessions or naturally through group interactions [46, 48].

Benefits could impact on other wider aspects of everyday life including health and employment [40, 41, 62, 84, 87] as well as having ripple effects on friends and family through the sharing of knowledge about social and cultural activities in local areas [78].

A range of potential mechanisms through which interventions were thought to bring about benefits were identified. Social network interventions provided the opportunities for distraction, allowing attendees to clear their minds which promoted self-reflection and the ability to process negative thoughts through engagement with valued activities [42, 62, 82, 86]. This led to calmer states which enabled cognitive and social skills to develop or be re-established [62, 86].

Social acceptance through connectedness with the local community helped individuals to see themselves in a more positive light, reminding them of their own strengths whilst challenging previously-held beliefs about what they could and could not do [48, 77, 80, 82]. One study found that that the use of humour around previously shame-inducing situations could support people to disidentify with negative identities and increase their sense of belonging [80]. This, combined with undertaking new or re-engaging with lost skills and pursuits, could engender a sense of pride and hope for the future [41, 45, 46, 77, 79, 89]. Studies also highlighted how connections made during intervention activities were considered to reduce the intensity of interactions within existing networks thereby improving social interactions more generally [45].

Participants described a virtuous cycle whereby participating in social network interventions developed skills and capabilities to support social connectedness, which in turn stimulated a sense of purpose, renewed interest in the world and desire for further social engagement and participation [39, 41, 46, 62, 77, 78, 81, 83, 86].

However, such benefits were not seen in all those who accessed social network interventions [40]. Involvement in interventions which were considered too challenging or encouraged downward social comparison had little impact on individual or social network outcomes [40].

#### The importance of a positive and safe space to support community integration

Participants expressed a strong desire to reduce social isolation and valued interventions that promoted community integration [42, 77, 87]. A key factor in the success of social network interventions was the context in which the intervention was delivered. Social network interventions were considered more likely to be successful if delivered in community venues external to formal health services. Those delivered in group settings were experienced as less intimidating as there was less pressure to make one-to-one connections [80].

Successful participation in real world activities was highly valued and indeed necessary for participants to benefit from social network interventions [39, 83]. Participants felt that interventions should be integrated into local communities and provide an access point to resources rather than further segregating people with mental health difficulties [80, 86]. However, such interactions could be challenging due to concerns about stigmatisation and previous negative experiences; facilitation or support to mitigate this was identified as imperative across studies [39, 48, 77, 78, 80]. This was particularly important at the early stages of involvement before trust and belonging had developed [80]. Engagement in shared activities that were not overburdensome (e.g. sport, games, shopping) helped to develop community relationships and overcome initial doubts and concerns [39].

Community engagement in non-judgemental settings had a range of benefits including increased community integration and improved connection to society more generally. These appeared particularly salient for those with serious mental health difficulties [78, 86, 89]. They also fostered the development of transferable skills that were easily integrated into everyday life and provided connections to wider society beyond the health care system [66, 78, 79, 82–84]. It was considered important to foster connections with people in the community who understood but did not necessarily have direct experience of mental health difficulties so that the focus of interactions was on shared interests or hobbies rather than ‘illnesses’ [40, 42, 48, 62, 84]. Self-selected, reciprocal and naturally occurring social connections were highly valued and considered more likely to occur outside of formal mental health settings [41, 42, 46, 81, 85, 86]. Participants also valued opportunities to help others and give back to the community [40].

Participants’ feeling safe, relaxed and accepted during intervention activities was considered instrumental to successful implementation of social network interventions. This was supported, where necessary, by home visits, particularly prior to community engagement [39–41, 46, 48, 79, 80]. These were more easily arranged for interventions in non-statutory settings, and particularly for nature or arts-based activities [42, 48, 62, 74, 77, 78]. Outdoor interventions were generally considered to be naturally restorative, calm, peaceful and safe which facilitated social interactions [48, 74, 86].

#### The need for available, accessible and sustainable activities

The availability of appropriate community resources for supported socialisation interventions and those interventions led by the third sector was raised as a challenge to the implementation of social network interventions in included studies [39, 83]. Funding for third sector activities was often precarious which meant that activities stopped with little notice. This was hard for intervention facilitators to keep abreast of and could be demotivating for participants [39]. Adequate staff training in relation to awareness of such activities locally and optimal ways to connect people to them was raised as a key facilitator to success.

Lack of accessibility to intervention activities was also highlighted as a barrier to intervention success. Issues included lack of funding for transport and access [39, 46, 67, 84], gender inaccessibility within activities [40, 41, 77], inflexibility which reduced accessibility for those with work or caring responsibilities [67, 78, 79, 89], stigma [41], lack of support for social anxiety and amotivation [42, 66, 67, 77, 79, 80, 82], social barriers, such as social norms and stereotypes [41], language barriers and low literacy [67, 77], rurality [67], and safety concerns created by location or timing of activities (e.g. at night) [78]. These barriers were particularly pertinent for participants who lacked family support to attend groups [41].

Insufficient consideration of accessibility issues could exacerbate health inequalities and meant that participants felt unable to realise their own potential for social connectedness [39, 84]. An example of particularly accessible environments was public allotments, which were considered widely available, inexpensive and inclusive settings, and as a result involvement was easier to maintain post intervention [86]. The provision of stipends was found to be a useful way to mitigate financial barriers [78].

Concerns were raised about the sustainability of certain activities and the potential impact of this on intervention participants [83]. Several studies highlighted harms caused by ending interventions without adequate consideration of how activities would be sustained [79, 87]. Most documented sustainability was attributed to participants’ planning for maintenance both within and outside their own networks [42, 78, 82]. This was considered particularly useful when facilitated as part of the intervention itself [46, 79], or where ongoing post-intervention engagement with activities or individuals was supported [62, 82, 87].

## Discussion

This systematic review and narrative synthesis aimed to identify and synthesise current evidence pertaining to the use of social network interventions for people with mental health difficulties, with a view to understanding their effectiveness, and the conditions in which these interventions might work best. Collectively, data from the 54 included studies demonstrated the utility of these types of interventions for people with mental health difficulties in terms of improving social networks and other health and social outcomes. Studies included a breadth of data and range of implementation and evaluation methods that lacked an explicit focus on context and outcome relationships. This made it difficult to draw definitive conclusion about what types of interventions work best for whom. However, we were able to identify conditions in which interventions can be optimally implemented.

In line with previous research, data supported the potential utility of interventions which focussed on supporting socialisation for people with mental health difficulties [12, 90, 91]. Most (21/54) studies included people with a range of mental health diagnoses. The remainder included participants with diagnoses of schizophrenia and/or psychosis (n = 12) or serious and/or enduring mental health difficulties (n = 10) with lesser attention given to other diagnoses. As a result, it was not possible to ascertain whom social network interventions worked best for. Encouragingly, participants demonstrated a strong desire for interventions which reduced social isolation and promoted community integration, suggesting high levels of acceptability across mental health conditions. Despite this, it is unlikely that social network interventions are a panacea, with the qualitative studies demonstrating the need to consider individual readiness for intervention participation and to ensure that interventions are sufficiently personalised to individual needs, preferences, heath, and access requirements [39, 83].

Factors that affected the implementation of social network interventions mirrored and extended those identified in the physical health field [23, 92]. In the current review, greater salience was given to the value of freedom, choice and personalisation within intervention activities, the need for individuals to be heard and progress at their own pace, and safe and non-judgemental spaces for intervention activities. Participants were more likely to raise concerns about stigma relating to mental health or past negative experiences with community organisations, which may relate to differences in the experience of mental health difficulties when compared to physical health difficulties [93]. Specific requirements relating to mental health and appropriate facilitation in this regard suggests a need for mental health specific training for intervention facilitators. Factors affecting the implementation of social network interventions appeared broadly applicable across mental health conditions and nuances in identified barriers and facilitators for people with specific diagnoses or severity were not discernible. Future research is required to ascertain whether there are condition-specific challenges to accessing social network interventions so that strategies to mitigate these can be developed.

This manuscript adds to existing literature by demonstrating the complexity of implementing social network interventions in the mental health field and identifying a range of access- related barriers which can hinder engagement. Failure to adequately consider the context in which an intervention will be delivered can exacerbate existing health inequalities by reducing access to potentially effective interventions [6]. This was evidenced across included studies; participants who were female, white, educated, married and had stronger baseline social networks and functioning were the most likely to access and benefit from these types of interventions [43, 57, 63]. This highlights the need for pre-implementation preparation to fully understand local delivery contexts and the needs of all those who might benefit from such interventions [94]. It is notable that only 2/54 included studies involved transgender participants and no included studies recorded the sexual orientation of study participants or considered neurodiversity. There were also limited numbers of identified facilitators in the included studies which related to issues of diversity and inclusion (Tables 3 and 4). This supports wider calls for prioritisation of equality, diversity and inclusion in the design, delivery and evaluation of social network interventions in future research in order to maximise intervention benefits [95]. This could be facilitated through co-production activities with those from diverse backgrounds and who represent or have insight into these communities.

This review identified a range of facilitators and barriers to implementing social network interventions in the mental health field potentially identifying a fundamental set of requirements as well as more bespoke requirements specific to type of need (Tables 3 and 4). Concomitantly, it highlighted the need to consider both downstream and upstream factors relating to implementation (i.e., individual motivation, capabilities, and opportunities and social and organisation level capacity). There was a particular tension between the sustainability of intervention activities and meaningful outcomes for participants. Consideration should therefore be given to how interventions are delivered (e.g. length of engagement time and potential enhanced role of facilitators) and the need to prioritise valued resources/activities that are sustainable [23].

In terms of implications for health services, findings illustrated the importance of targeting people with lower levels of baseline social functioning and people with smaller social networks or networks of poorer quality at baseline [57, 63]. Given the need for a safe and accessible venue for intervention delivery and the importance of the facilitator role, provision for someone to accompany people to activities, especially during early interactions should be included in intervention design [80]. The findings also lend support to recent calls to reorient mental health service provision and reduce the focus on individual psychopathology and one-to-one interactions with health professionals [96, 97]. An alternative focus oncare provision through outreach work and engagement with community resources, to harness the collective value of social networks would potentially be of more value [98]. This review also highlights the need to prioritise third sector funding to provide suitable resources for people to access [29].

### Strengths and limitations

This review benefits from comprehensive search strategies which incorporated both published and grey literature, the inclusion of papers published in languages other than English, and the rigour of screening and extraction processes. Hand-searching of relevant journals and included papers identified a further two papers to be included. Another strength was the inclusion of seven members of the review team who had lived experience of mental health difficulties and two members who had clinical experience of delivering mental health care. This enhanced the review in several ways: ensuring that search terms were inclusive and comprehensive; clarifying understanding of social network interventions; and enhancing contextualisation of implementation barriers and facilitators. The qualitative studies provided most learning in relation to the use of social network interventions for people with mental health difficulties. There is a need to further this research by testing these factors against outcomes through powered mechanistic trials.

Several limitations should also be considered. First, incorporation of two grey literature databases is unlikely to have fully addressed potential publication bias. Second, whilst attempts were made to integrate study quality into the narrative synthesis, the overall quality of included studies may have impacted on the synthesis presented. Finally, the review only included the views of participants in social network interventions in relation to perceived barriers and facilitators to implementation, and it may be that these participants were not fully aware of all the potential factors that impacted implementation. The review also did not include those who had a primary diagnosis of substance misuse, autism spectrum disorders, dementia, attention deficit hyperactivity disorder (ADHD), or cognitive impairment. These limitations should be carefully weighed against the feasibility of managing and synthesising manuscripts from a review strategy that was more inclusive.

## Conclusion

Strategies for improving the social networks of people with mental health difficulties should focus on ensuring access to personalised and supported social activities outside of formal mental health services. To optimise access and uptake, accessibility barriers should also be carefully considered within implementation contexts, and equality, diversity and inclusion should be prioritised in intervention design, delivery and evaluation, as well as in future research in this area.

## Electronic supplementary material

Below is the link to the electronic supplementary material.

Supplementary Material 1

Supplementary Material 2

Supplementary Material 3

## Data Availability

All data generated or analysed during this study are included in this published article [and its supplementary information files].

## Footnotes

1 Personally-meaningful communities, connections and ties which link people to relationships, resources and activities that may help to manage and optimise their mental health [11].

2 Terms are used that were included in the original paper.

## List of abbreviations

ADHD: Attention deficit hyperactivity disorder
MMAT: Mixed Methods Appraisal Tool
PICO: Population, intervention, comparatory, outcome
PPI: Patient and public involvement
PRESS: Peer Review of Electronic Search Strategies
PRISMA: Preferred Reporting Items for Systematic Reviews and Meta-Analysis
RCT: randomised control tried
UK: United Kingdom
