# Supplementary figures and images for: Exploring the use of social network interventions for adults with mental health difficulties: a systematic review and narrative synthesis
Source: BMC Psychiatry. 2023 Jul 7;23:486. doi: 10.1186/s12888-023-04881-y (PMC10329398; doi:10.1186/s12888-023-04881-y)

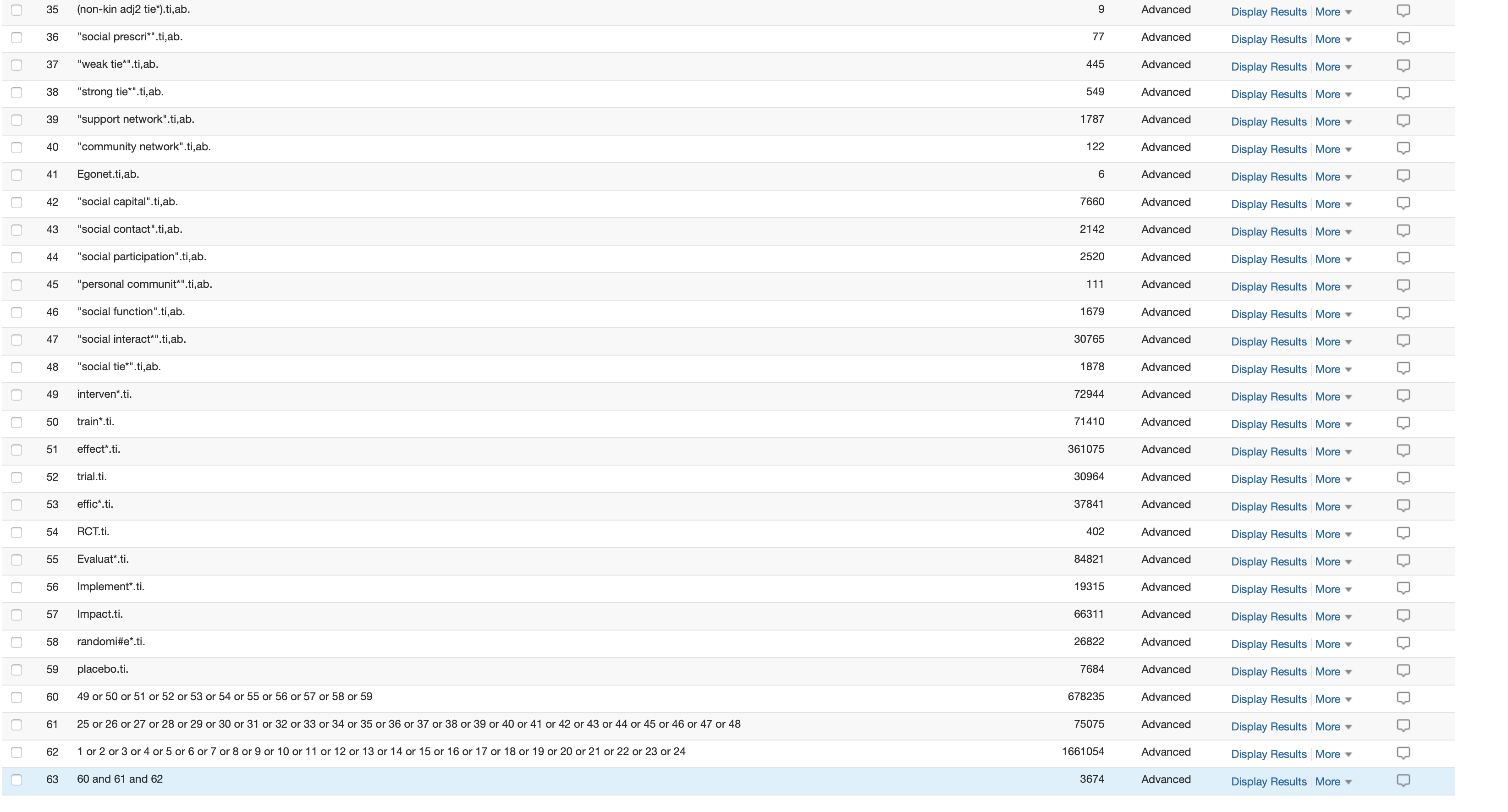

Supplement: Supplementary file 2 — Supplementary Material 2 [file 12888_2023_4881_MOESM2_ESM.docx]
