# Supplementary material for: Exploring the use of social network interventions for adults with mental health difficulties: a systematic review and narrative synthesis
Source: BMC Psychiatry. 2023 Jul 7;23:486. doi: 10.1186/s12888-023-04881-y (PMC10329398; doi:10.1186/s12888-023-04881-y)
Supplement: Supplementary file 3 — Supplementary Material 3 [file 12888_2023_4881_MOESM3_ESM.docx]

**Table S1: Context**

| **STUDY ID** (Author last name, year) | **COUNTRY** | **STUDY DESIGN** | **RECRUITMENT METHOD** | **RECRUITMENT SETTING** | **DELIVERY SETTING** | **DATA COLLECTION** | **FUNDING** | **INTERVENTION TYPE (type, components and relevant aims)** | **INTERVENTION LENGTH** |
| --- | --- | --- | --- | --- | --- | --- | --- | --- | --- |
| 1. Abotsie., 2021 | UK | Mixed methods | Not reported. | Not reported. | Community. | Self-complete questionnaire. | The Burdett Trust for Nursing & Norwich Clinical Commissioning Group. | **Type of intervention:** Supported social/community activity.  **Components:**  (1) Hour long football matches  (2) Drop-in sessions from psychologists, mental health nurses and peer workers with mental health experience.  **Aim:** To promote social inclusion and social wellbeing. | Open-ended, weekly sessions. 12-week measurement from baseline to outcome. |
| 2. Aggar., 2021. | Australia | Other quantitative | GP invitation. | GP surgery. | Community. | Questionnaires administered by social workers | Primary and Community Care Services,  NSW, Australia. | **Type of intervention:** Supported social/community activity.  **Components:**  (1) Weekly arts and crafts group led by artist and co-facilitated by a mental health social worker.  **Aim:** To increase social participation. | 2–3 hours per week for 10 weeks |
| 3. Ammerman, 2013 | USA | RCT | Referral by health professional. | Referral by home visitor. | Home. | Standardised outcome measures. | National Institute of Mental Health. | **Type of intervention:** One-to-one cognitive behavioural therapy  **Components:**  (1) In-home cognitive behavioural therapy delivered by two licenced master’s level social workers.  **Aim:** To improve social networks and social functioning. | 15 weekly sessions of 60 mins plus a booster session one-month post-treatment. |
| 4. Bailey, 2020 | Australia | Other Quantitative | Not reported. | A tertiary-level outpatient mental health service that is part of a state government-funded youth mental health service. | Online. | Self-report measures. | Mary Elizabeth Watson Early Career Fellowship in Allied Health from the Royal Melbourne Hospital, with additional support from Future Generation Global. | **Type of intervention:** Peer support (online).  **Components:**  (1) Newsfeed where participants and moderators can post comments, information, upload pictures and videos, and reply to content posted by other user  (2) An online problem-solving forum. Group members can offer solutions. All content moderated by peers.  **Aim:** To improve social connectedness. | The platform was live for seven months. |
| 5. Becker, 1998 | England | Other Quantitative | Case identification exercise in two catchment areas from a range of community and hospital resources. | A range of community and hospital resources. | Community mental health service. | Structured interviews. | Author supported by a Lynen grant of the Alexander von Humboldt-Stifung, Bonn, FRG. | **Type of intervention:** Intensive/enhanced community treatment.  **Components:**  (1) Two specialised intensive teams were set up.  **Aim:** To improve social networks. | Not reported. |
| 6. Bertotti, 2018 | UK | Mixed methods. | 7 participants were a random sample selected from service users referred by their GP. 10 were selected with the help of social prescribers. | GP surgery; social prescriber contact. | GP surgery initially, then in the community. | Interviews (one-to-one, both face-to-face and telephone) with open ended questions; and observations. | Health Foundation (Shine Award) | **Type of intervention:** Supported social/community activity.  **Components:**  (1) Wellbeing plans created with social prescribers who then referred participants to community organisations  **Aim:** To reduce social isolation.  . | Up to six 40-minute sessions. |
| 7. Bitter, 2017 | Netherlands | RCT | Information sheet sent to current service users. Followed up by researcher or via the staff for participation in the study. | Three organizations for sheltered and  supported housing. | Three organizations for sheltered and  supported housing. | Self-reported questionnaires. | Funded by five organizations (Kwintes, RIBW Arnhem and Veluwevallei, RIBW Fonteynenburg, RIBW K/AM and RIBW Gooi- and Vechtstreek,and Fonds Storm Rehabilitatie) for sheltered and supportive housing and Storm Rehabilitation. | **Type of intervention:** Intensive/enhanced community treatment – rehabilitation programme.  **Components:**  (a) Comprehensive approach to rehabilitation training for professionals. Professionals trained in a specific recovery model to support and enhance their usual work with clients.  **Aim:** Improve quality of a participant’s social environment. | Not reported. |
| 8. Bjorkman, 2000 | Sweden | Other Quantitative | Recruitment from psychiatric services and social services, and by self-referral. | Recruitment from psychiatric services and social services, and by self-referral. | Statutory health services. . | Case managers made weekly reports and clients were assessed using various measures. | The Swedish National Board of Health and Welfare and the VaÊrdal Foundation. | **Type of intervention:** Intensive/enhanced community treatment – case management.  **Components:**  (1) Ten new case management services.  **Aim:** To improve social networks. | Not reported. |
| 9. Bradshaw, 1998 | UK | Qualitative | Recruited via befriending scheme co-ordinator. | Not clearly reported but recruitment via befriending scheme. | In the community and in the participants' homes. | Semi-structured interviews. | None reported. | **Type of intervention:** Peer support (befriending)  **Components:**  (1) People with long-term mental health problems matched with volunteer befriender  **Aim:** To improve social support obtained from social networks. | 1-9 months (modal time period 1-3 months). The time spent by volunteers with subjects ranged from 1 to 4 hours, the modal contact time was 1-2 hours. |
| 10. Bragg, 2014 | UK | Mixed Methods | Recruited by project worker of established nature-based projects for mental health. | Nature-based projects at nine sites. | Community. | Questionnaire with rating scales and open narrative questions. | Big Lottery Fund. Projects evaluated were funded by various sources including community mental health teams, local authorities, and the third sector. | **Type of intervention:** Supported social/community activity.  **Components:**  (1) 130 environmental projects for people living with mental health problems to become involved in green activities to improve confidence, self-esteem, and their physical and mental health.  **Aim:** To increase social inclusion. | 8 weeks to 4 years. Mean length of time over all projects was 6 months. |
| 11. Calsyn, 1998 | USA | RCT | Not reported. | Emergency shelters/health services/A&E. | Community. | Interviews by master’s level psychologists and social workers. | National Institute of Mental Health. | **Type of intervention:** Intensive/enhanced community treatment.  **Components:**  (1) 24-hour emergency services.  (2) Indefinite care periods.  (3) Comprehensive services including benefit support and support with daily living activities.  (4) Home delivery.  (5) Team approach.  **Aim:** To improve the social relationships of attendees. | Unspecified, but study ran for 21 months. |
| 12. Castelein, 2008 | The Netherlands | RCT | Not reported. | Mental Health Care Centres. | Mental Health Care Centres. | Independent questionnaire completion with a professional available to support. | Zon Mw (the Netherlands Organisation for Health Research and Development), the Rob Giel Research Center, and The Roos Foundation. | **Type of intervention:** Peer support (closed peer support group).  **Components:**  (1) Closed peer support group (10 patients).  (2) Participant led topics for sessions.  (3) Pre-defined structure for sessions.  (4) Facilitated by a nurse.  **Aim:**  To improve social networks. | 8 months. 16 sessions of 90 min biweekly over 8 months. |
| 13. Chang, 2016 | China | RCT | Follow-up study. | Statutory health services. | Statutory health services. | Interviews with patients and relatives, histories, medical records. | Commissioned Research on Mental Health Policy and Services (SMH-29) of the Food and Health Bureau, the Government of Hong Kong Special Administrative Region. | **Type of intervention:** Intensive/enhanced community treatment.– case management  **Components:**  (1) A tailored mental health service for first-episode psychosis with a phase-specific case management approach.  **Aim:** To improve social relationships. | One-year extension to current two-year program. |
| 14. Chowdhary, 2016 | India | Mixed Methods | Referrals from general practitioners and psychiatrists, and self-referrals. | Not reported. | 11 primary health centres (later adapted to be delivered at home in response to patient feedback). | Focus group discussions with counsellors; in-depth interviews with supervisors and patients.  Assessments were carried out by evaluators | Wellcome Trust Senior Research Fellowship. | **Type of intervention:** One-to-one intervention (behavioural activation).  **Components:**  (1) A brief structured psychological treatment for moderate/severe depressive symptoms that is delivered by lay counsellors in primary care.  **Aim:** To increase activation of social networks. | Delivered in three phases with an optimum of six sessions (up to a maximum of eight), each session lasting 30–40 min, at weekly or fortnightly intervals. |
| 15. Darongkamas, 2011 | UK | Mixed Methods | Members of the football club were invited to participate. | Community football club. | Community football club. | Questionnaires and semi-structured interviews. | None reported. | **Type of intervention:** Supported social/community activity  **Components:**  (1) Men’s community football club.  **Aim:** To reduce social isolation | Not clearly reported. Most players had been participating for between eight months and the three years over which the scheme had run. |
| 16. Felton, 2009 | UK | Qualitative | Recruited by unit staff. | Seven community-based residential mental health rehabilitation units. | Seven community-based residential mental health rehabilitation units. | Photography to gather information from those being supported by rehabilitation services. Participants produced narrative to accompany images. | None reported. | **Type of intervention:** Other  **Components:**  A social inclusion network was developed to co-ordinate a small participatory project that  examined the experiences of inclusion in the community from the perspectives of those using and working in rehabilitation adult mental health services in one locality.  **Aim:** To understand and promote social inclusion. | Not reported. |
| 17. Fieldhouse, 2003 | England | Qualitative | Recruited by manager of the community mental health team. | Community mental health team horticultural allotment group. | Community mental health team horticultural allotment group | Semi-structured interviews and focus groups. | Thrive. | **Type of intervention:** Supported social/community activity.  **Components:**  (1) Horticultural allotment group established.  (2) Service users referred from inner city community mental health team.  **Aim:**  To improve social networks. | Not reported. |
| 18. FitzGerald, 2011 | UK | Other Quantitative | Recruited by occupational therapists. | Four long-stay units in a low-secure, rehabilitation forensic service. | Four long-stay units in a low-secure, rehabilitation forensic service. | Quantitative outcome measures collected by occupational therapists who also ran the groups. | None reported. | **Type of intervention:** Supported social/community activity.  **Components:**  (1) The programme entailed graded community engagement and one-to-one goal planning with a unit-based occupational therapist, in addition to normal treatment.  **Aim:** To improve social functioning | No set number or sessions or prescribed time period. |
| 19. Fowler, 2018 | UK | RCT | Potential participants were approached by their care  coordinator at the early intervention service. | Four specialist early intervention services. | Four specialist early intervention services. | Research assistants visited participants at home to undertake assessments. | National Institute for Health Research. | **Type of intervention:** Intensive/enhanced community treatment.– case management + CBT  **Components:**  (1) Case management to encourage socially withdrawn individuals back into social environments  (2) CBT to promote engagement and overcome the symptoms that impede it.  **Aim:** To promote social recovery.  . | Approx. 21 months. |
| 20. Friedrich, 2018 | England | Qualitative | Recruited by occupational therapist. | Not reported. | Community sport venues. | Semi-structured interviews. | NIHR School for Public Health Research (SPHR) Public Health Practice Evaluation Scheme (PHPES). | **Type of intervention:** Supported social/community activity.  **Components:**  (1) Football intervention, delivered jointly between mental health practitioner and coaches from the football club.  **Aim:** To improve social inclusion and integration. | Not reported. |
| 21. Garety, 2006 | England | RCT | Admissions to hospital and all new referrals to outpatient sector teams were screened to identify potential cases. | Statutory health services. | An NHS service for first episode psychosis. | Standardised assessments by trained independent research staff. | Directorate of Health and Social Care for London R&D rate of Health and Social Care for London R&D Organisation and Management Programme. | **Type of intervention:** Intensive/enhanced community treatment.  **Components:**  (1) A tailored NHS outpatient mental health service for first-episode psychosis.  (2) A single point of access for all mental health and social welfare needs.  (3) Specially adapted early intervention and CBT interventions tailored to individual need.  **Aim:** To resume leisure pursuits and retain or re-establish supportive social network | Not reported, but data collected over 18 months. |
| 22. Gater, 2010 | England | RCT and qualitative data. | Multilingual invitation posters were displayed to encourage recruitment. | GP surgeries. | Community centre. | Quantitative questionnaires, feedback form and focus groups. | Medical Research Council. | **Type of intervention:** Supported social/community activity.  **Components:**  (1) Culturally sensitive intervention combining group activities and psychoeducation  (2) Facilitated groups at a local community centre for weekly sessions over 10 weeks.  (3) In the first session, the participants choose from a list of indoor and outside activities for subsequent sessions.  (4) psychoeducation session provided information about depression including its nature, symptoms, causes and treatment.  **Aim:** To develop informal networks that engage women in social contacts, and link them to appropriate treatment. | 10 weekly sessions. |
| 23. Hacking, 2008 | England | Other Quantitative | Not reported. | Not reported. | Statutory services. | Routinely collected data. | None reported. | **Type of intervention:** Other  **Components:**  (1) Tool to facilitate collaboration between the service user and the practitioner.  (2) Form of action research.  (3) Provides a map of the person’s personal network of places and relationships and when repeated allows patient and practitioner to monitory and change together.  **Aim:** To promote social inclusion. | Not reported. |
| 24. Hanlon, 2019 | Scotland | Qualitative | Recruited through an ongoing RCT. | GP surgeries. | GP surgeries. | Semi-structured interviews. | NHS Health Scotland. | **Type of intervention:** Supported social/community activity.  **Components:**  (1) One-to-one work to refer and support use of community resources.  (2) Awareness events relating to community resources.  (3) Shared learning events with practices and community organisations  **Aim:** To improve wellbeing by connecting patients to appropriate community resources | Varied: from one session to continuing over several months. |
| 25. Hanly, 2020 | Australia | Qualitative | Not reported. | Not reported. | Community settings. | Online reflection and semi-structured interviews. | William Buckland Foundation, the Ian Potter Foundation and John T Reid Charitable Trusts. | **Type of intervention:** Peer support  **Components:**  (1) Activities for sessions were planned collaboratively with individually tailored recovery goals  (2) Activities included daily living tasks, practicing social interactions involving food, and making connections in the community.  (3) Mentors were encouraged to share their own story of recovery to foster hope and provide empathic support  **Aim:** To build social networks  . | 13 three-hour sessions over a three-to-six-month period (i.e. some pairs met weekly, while others met fortnightly) |
| 26. Haslam, 2016 | Australia | Other Quantitative | Not specified but via university psychology clinic. | University psychology clinic. | University psychology clinic. | Self-complete questionnaires. | Canadian Institute for Advanced Research Social Interactions, Identity and Well-Being Program. | **Type of intervention:** Supported social/community activity + psychoeducation  **Components:**  (1) Psychoeducation programme delivered in groups, designed to give people the knowledge and skills they need to manage their social group memberships.  (2) Five modules, each containing a series of exercises and discussions.  (3) Module 5 involves developing and implementing social plans.  **Aim:** To improve social connectedness | 8-9 weeks. First 4 modules delivered weekly and the final a month later, each taking between 60 and 75 min to deliver. |
| 27. Haslam, 2019 | Australia | RCT | Self and clinician referrals: recruitment notices were distributed via letters and flyers. | Community and university services (e.g., GPs, psychologists, counsellors, psychology interns, and other health professionals). | Psychology clinic at university. | Participants completed baseline measures and follow-up measures. | None reported. | As Haslam 2016. | 4 weekly sessions, lasting 60-90mins, with a 5^th^ session a month later. |
| 28. Hassan., 2020 | UK | Qualitative | Purposive sampling – email invitation. | Community service provided by NHS - The Life Rooms | Community service provided by NHS - The Life Rooms | Focus Groups. | This project was funded by The National Institute for Health Research  Collaboration for Leadership in Applied Health Research and Care North  West Coast (NIHR CLAHRC NWC). | **Type of intervention:** Supported social/community activities.  **Components:**  (1) Social prescribing.  (2) The Life Rooms provides learning opportunities, delivering free courses covering a range of topics such as wellbeing, understanding and management of mental distress, social factors related to mental distress and other social and creative offerings.  **Aim:** To address social determinants of mental health | Open ended. |
| 29. Howarth, 2018 | UK | Mixed Methods | Purposive sampling. | Not reported. | Outdoor, green space provided by a social enterprise. | Quantitative data from participants’ ‘mental health recovery stars’. Semi-structured focus groups and interviews. | Big Lottery Reaching Communities Fund. | **Type of intervention:** Supported social/community activities.  **Components:**  (1) Person-centred, asset-based outdoor therapeutic horticulture programme.  **(**2) Provides a simple guide to growing and enables volunteers to participate in sowing, growing and harvesting of products  **Aim:** To improve social inclusion and engagement. | Not clearly reported. Data collection over 2-year period but no record of how long participants attended the programme. |
| 30. Johnson, 2018 | England | RCT | Referral by health professional. | Crisis resolution team caseloads. | Community. | Questionnaires delivered by trained researchers. | National Institute for Health Research under its Programme Grants for Applied Research programme. | **Type of intervention:** One-to-one recovery focussed intervention – peer led.  **Components:**  (1) One-to-one recovery focussed work to support community integration and social network enhancement by peer workers.  (2) Peers offered supportive listening and instilled hope through appropriate sharing of skills and coping strategies acquired in their own recovery.  (3) Activities focussed on a recovery workbook.  **Aim:** To improve community integration and enhance social networks. | Ten individual sessions of 1 hour each with a peer support worker for four months. |
| 31. Kaltman, 2016 | USA | Mixed Methods | Flyers, referral by clinic staff, and outreach screening in the waiting room of a community primary care clinic. | A community primary care clinic serving low-income, uninsured patients. | A community primary care clinic serving low-income, uninsured patients. | Baseline and post-intervention evaluations, and a semi-structured interview. | National Institute of Mental Health. | **Type of intervention:** Peer support + psychoeducation.  **Components:** An intervention which consisted of both individual and group sessions, combining various therapeutic approaches to reduce symptoms of depression and PTSD and increase social support.  **Aim:** To increase social support received by social networks. | Individual component: 90 minutes that could be delivered either in two or three sessions. Group component: five 90-minute sessions. |
| 32. Lund, 2019 | Sweden | Qualitative | Recruited by occupational therapists. | Clinic and community settings. | Outpatient psychiatry and community mental health settings. | Interviews. | Swedish Research Council. | **Type of intervention:** Intensive/enhanced community treatment.– occupational therapy incorporating peer support..  **Components:**  (1) A manualised, group-based occupational therapy intervention that aims to provide a supportive structure and informal peer-support learning environment.  (2) Participants set personal goals related to each week’s topic and had self-assigned homework.  **Aim:** To improve activity engagement, functioning and well-being | 12-week course with additional booster sessions at weeks 14 and 16. Meetings took place once a week and lasted for 1.5–2 hours including a short coffee break. |
| 33. Mak, 2016 | China | Mixed Methods | Non-governmental organisation staff at various service units. Matched controls were identified from the same service units of the organisation. | Large non-governmental organisatio. | Not reported but seems to have been held on the premises of these services. | Assessments were conducted with the participants. | Social Welfare Development Fund of the Social Welfare Department, Hong Kong SAR. | **Type of intervention:** Peer support  **Components:**  (1) Consumer-led illness self-management recovery programme delivered in a group setting.  (2) Creating a daily plan which includes the activities that help to stay healthy.  (3) Peer support  **Aim:** to assist individuals with mental illness to develop a range of effective strategies for self-directed care | Eight 1.5-hour sessions. |
| 34. Margrove, 2013 | UK | Mixed Methods | People on a waiting list for an introductory course across four localities were invited to take part. Advertised publicly through mental health services. | In the community and mental health services. | Community venues providing Open Arts courses. | Self-complete questionnaires and focus group discussions. | South Essex Partnership University NHS Foundation Trust. The Faculty of Health, Social Care and Education at Anglia Ruskin University provided additional funding for the qualitative work. | **Type of intervention:** Supported community/social activity.  **Components:**  (1) Art groups in community venues for people with mental health needs.  (2) Sessions include various types of visual media.  **Aim:** To promote wellbeing and social inclusion. | 12 weeks on one day each week. |
| 35. Mathias, 2019 | India | Qualitative | Door-to-door recruitment. | Not reported. | Community and health. | In-depth interviews and focus group discussions. | Disability Research Initiative, University of Melbourne, and the Australia India Institute Project. | **Type of intervention:** Supported community/social activity + psychoeducation.  **Components:**  (1) Peer facilitators delivered an emotional resilience psychoeducation programme  (2) Participants were encouraged participants to participate in collective activities and supported to access other services.  **Aim:** To promote social inclusion. | 17 sessions over 6 months. |
| 36. Mazzi, 2018 | Italy | Other Quantitative | Not reported, | Not reported. | Not reported. | Case Register of the Mental Health Department integrated with additional information gathered from the patients’ clinical notes | None reported. | **Type of intervention:** Supported community/social activity  **Components:**  Social inclusion program, including coaching and supported socialisation.  (1) Participants are assessed and supported to reflect on their interests and preferences for activities.  (2) Workers provide coaching before activating the local community network and resources  (3) Plans are revised and reassessed.  **Aim:** To increase social inclusion and promote active social participation. | The median duration of program was 548 days. During this period, the median number of social inclusion activities per patient was 4. |
| 37. O'Brien, 2011 | England | Qualitative | Recruited by health or social care practitioner or GP, or by self-referral. | Not reported. | Therapeutic garden. | Ethnographical case study was preceded by 10 interviews. | Forest research, the research agency of the Forestry Commission in Britain, undertook the general environmental  volunteering research in partnership with Deakin University, Australia. The work was part funded by the Scottish Forestry Trust and the Forestry Commission. | **Type of intervention:** Supported community/social activity  **Components:**  Volunteering at a therapeutic garden.  **Aim:** To promote social inclusion. | Participants would volunteer at Meanwhile on average two to three times a week for a full day over a number of months, depending on the individual. |
| 38. O’Connell, 2020 | USA | RCT | Recruited during admission to hospital. | Inpatient mental health unit. | Combination of inpatient mental health unit and outpatient clinic, with home visits following discharge and support groups after that. | Self-reported outcome measures with additional data on inpatient and outpatient service use drawn from the system’s administrative database. | National Institute on Drug Abuse. | **Type of intervention:** Supported community/social activity and peer support  **Components:**  (1) Social skills training  (2) Social engagement with included peer support. twice-weekly mutual support groups as well as social and recreational outings.  **Aim:** To increase social connections and engagement in self-care | Skills training open-ended. Social engagement program ran for 3 months following discharge from hospital. |
| 39. Petryshen, 2001 | Canada | Other Quantitative | All eligible patients referred to the programme. | Community health centre. | Community health centre. | Questionnaires and Staff main­tained daily contact records. | Service funded by Onatorio Ministry of Health. No information about research funding. | **Type of intervention:** Intensive/enhanced community treatment.– social recreation component  **Components:**  A multi-level, multi-strategy process is applied to facilitate the process of relationship development. Program delivery is directed at two levels:  (1) individual – skills based and encouragement of social participation  (2) social or environmental – promoting social network building.  **Aim:** To help individuals establish and maintain satisfactory social bonds. | The intervention itself has no specified length. |
| 40. Rivera, 2007 | USA | RCT | Recruitment in inpatient units by researchers. | Inpatient units. | Combination of clinic-based service provision and that provided in the general community by peer workers. | Assessment of hospital records, monthly interviews using self-report instruments. | New York City Department of Health and Mental Hygiene, the New York State Office of Mental Health, and the New York City Health and Hospitals Corporation. | **Type of intervention:** Supported community/social activity - peer led  **Components:**  (1) Peer workers supported clients in social activities and developed supportive social networks among clients.  (2) one-to-one and group social activites were undertaken in and around clients’ homes and community locations.  (3) Clinic based care.  **Aim:** To improve social networks. | Unclear but 12 months follow-up period. |
| 41. Segal & Holschuh, 1991 | USA | Other Quantitative | Not reported. | Not reported. | Not reported. | Structured interviews, survey, and obtaining hospital records. | National Institute of Mental Health and the Robert Wood Johnson Foundation | **Type of intervention:** Other – enhanced social accommodation.  **Components:**  (1) Supportive, transitional, high-expectation sheltered care environment.  **Aim:** To improvs social networks. | Not reported. |
| 42. Sexton, 1992 | England | Mixed Methods | Recruited by staff at a community mental health day facility. | Community mental health day facility. | Community mental health day facility. | Structured interviews. | None reported. | **Type of intervention:** Intensive/enhanced community treatment – community mental health day faciltiy  **Components:**   1. Community mental health day facility. 2. No time limit. 3. An occupational therapist designed activities to provide opportunities for self-development, for social interaction and to increase self- confidence   **Aim:** To improve social networks. | Not time limited. |
| 43. Sheridan, 2015 | Ireland | RCT | Circulation of promotional materials and referral directly from mental health services. | Referral from mental health services and self-referral. | Community. | Structured data collection interviews - quantitative. | Health Research Board. | **Type of intervention:** Supported community/social activity – volunteer led.  **Components:**  (1) Participants matched with volunteer  (2) asked to engage in social/leisure activities for 2 hours weekly over a 9-month period.  (3) Provided with a stipend of 20 euros per month.  **Aim:** To improve social functioning | 9 months. Two hours per week. |
| 44. Sheridan, 2018 | Ireland | Qualitative | Recruited using information sessions delivered in centres connected to each mental health service or by mental health practitioners approaching service users individually. | Statutory services. | Within five discrete Irish mental health services. | Participants completed diaries to reflect on the process of engaging in supported socialisation and to describe how they experienced  taking part in the programme. | Health Research Board. | **As Sheridan 2015.** | Social/leisure activities for approximately 2 hours each week over a 9-month period. |
| 45. Snethen, 2012 | USA | Mixed Methods | Recruited via ACT [Assertive Community Treatment] team case managers. | Not reported. | Community. | Structured interview to confirm diagnosis, an initial semi-structured interview, nine modified day-reconstruction methods interviews and a final exit interview. | None reported. | **Type of intervention:** Supported community/social activity  **Components:**  A recreational-therapy intervention modelled after the individualised placement and support model, to support community-based recreation participation  (1) Matching clients with interest-based activities  (2) promoting autonomy and providing onsite training through co-participation.  **Aim:** To increase community participation. | 10 weeks. |
| 46. Suto, 2021 | Canada. | Qualitative. | Flyers and invitation letters. Word of mouth aided ongoing recruitment. | Health service and supported housing sites. | Community. | Semi-structured interviews, participant observations, focus groups, two mapping activities. | This research was supported by a grant from the Vancouver Foundation (UNR13-0037). | **Type of intervention:** Supported community/social activity  **Components:**  (1) Horticultural therapy - These sessions combined education on edible gardening with social interaction, and peer-to-peer and facilitator-led learning  **Aim:** To improve wellbeing and social connectedness.  . | 3 years. |
| 47. Tarbet, 1985 | USA | Qualitative | Researcher was involved with the intervention group already so asked to be participant observer to conduct the study. | Self-help group held in a patients’ rights office in the community. | Self-help group held in a patients’ rights office in the community. | Semi-structured interviews, supplemented by participant observation. | Doctoral study. | **Type of intervention:** Peer support – self-help support group  **Components:**   1. Weekly support meetings. 2. Supplementary advocacy meetings twice a moth   **Aim:** To improve social networks.  . | Effects were examined over a period of minimum two months to maximum two years. |
| 48. Tempier, 2012 | England | RCT | Not reported. | Early onset trial. | Community. | Questionnaires administered by trained researcher. | None reported. | **Type of intervention:** Intensive/enhanced community treatment  **Components:**  (1) Assertive community treatment  **Aim:** To increase social network size and support provided by social networks. | 12 months. |
| 49. Terzian, 2013 | Italy | RCT | Health professional at community mental health service. | Community mental health services. | Community. | Psychiatrist assessment. | Educational grant of the Consorzio Mario Negri Sud, an independent public–private research institute, which contributed the facilities for data collection and handling, as well as the services for data quality monitoring through its certified unit. | **Type of intervention:** Supported community/social activity  **Components:**  (1) Identification of potential social activities.  (2) Access to and engagement with activities supported by health professionals or natural volunteers.  **Aim:** To improve social networks. | 3-6 months. |
| 50. Thorup, 2006 | Denmark | RCT | Referral by health professional. | Health services. | Community. | Structured interviews and assessments by independent, trained professionals. | The Danish Ministry of Health, the Danish Ministry of Social Affairs, the Danish Medical Research Council, Copenhagen Hospital Corporation, Aarhus County, the Wørzner Foundation and the University of Copenhagen. | **Type of intervention:** Intensive/enhanced community treatment – assertive community treatment.  **Components:**  (1) Assertive community treatment  (2) social skills training  (3) multi family groups.  **Aim:** To improve social networks. | 24 months. Took place biweekly in the first 2 months and then once a week in the following 10 months. |
| 51. Van der Venter, 2014 | UK | Mixed Methods | Not reported. | Not reported. | Inner city GP practices or community centres. | Assessment measures and interviews with a topic guide. | South West Public Health Training Programme. | **Type of intervention:** Supported community/social activity  **Components:**  (1) Arts-on-referral scheme, where individuals are referred to regular art groups.  **Aim:** To improve wellbeing. | 20 weeks. |
| 52. Varga, 2018 | Hungary | RCT | Not reported. | Community-based mental health care institution. | Community-based clubs. | Participants were assessed by researchers on all study variables at baseline and follow up. | National Brain Research Programme. | **Type of intervention:** Intensive/enhanced community treatment – community clubs.  **Components:**  (1) Community clubs offering skills training, psychoeducation, group activities, socialisation, and continuous access to mental health care during the day.  **Aim:** To improve social functioning | Six-month treatment period. |
| 53. Webber, 2019 | UK | Other Quantitative | Social media and UK Mental Health Research Network. | Health and social care agencies working with adults with mental health problems or a learning disability in England. | Health and social care agencies working with adults with mental health problems or a learning disability in England. | Interviews. | National Institute for Health Research School for Social Care Research. | **Type of intervention:** Supported community/social activity  **Components:**  (1) Connecting People’s eight-step process involves a worker exploring an individual’s existing connections with them; exploring new opportunities for engagement in activities, groups, networks, clubs, societies or resources in the individual’s local community; developing an action plan and sourcing appropriate support for them to access their community; addressing barriers to social and community engagement; and reviewing progress towards achieving social goals.  **Aim:** To improve social capital, social inclusion and wellbeing.  . | Practitioners are expected to use the model to inform their daily practice with all service users. |
| 54. Webber, 2021 | UK | Other quantitative. | Potentially eligible participants were provided with study information sheets by their care co-ordinator or a Clinical Studies Officer in participating teams. | Statutory health services. | Mental Health Trusts. | Participants were interviewed by telephone using a standardised interview | Funded by the National Institute for Health Research School for Social Care Research (ref C088/CM/UYYB-P114). | As Webber 2019. | Not reported. |

**Table S2: Participants.**

| **STUDY ID (Author last name, year)** | **N RECRUITED** | **AGE RANGE (years)** | **MEAN AGE (years)** | **ETHNICITY** | **GENDER  % Female** | **PRIMARY DIAGNOSIS OF TARGET POPULATION** |
| --- | --- | --- | --- | --- | --- | --- |
| 1. Abotsie,2021 | 142 | 21-42 | Not reported. | Not reported. | 0% | Self- reported emotional distress. |
| 1. Aggar, 2021. | 24 | Not reported. | 45 | Not reported. | 69% | All mental health problems. |
| 1. Ammerman, 2013 | 93 | 16–37 | 21.9 | White: IH-CBT: 30 (63.8%); SHV: 28(60.8%)  African American: IH-CBT: 14(29.9%); SHV: 16(34.8%) Native American: IH-CBT: 1(2.1%); SHV: 0 (0.0%) Native Hawaiian or other Pacific Islander: IH-CBT 1(2.1%); SHV: 1(2.2%) Bi-racial: IH-CBT: 1(2.1%); SHV: 1(2.2%) Latina: IH-CBT: 3(6.4%); SHV: 4(8.7%)  None: IH-CBT: 44(93.6%); SHV: 42(91.3%) | 100% | Major Depressive Disorder (MDD) |
| 1. Bailey, 2020 | 23 | 16-25 | 21.7 | Not reported. | 55% | Complex depression. |
| 1. Becker, 1998 | 210 | Not reported. | 40.5 | 62% (n=80) were White. Of the 49 non-white participants 37 were of Black Caribbean and 7 of Black African origin. | 45 | Psychosis. |
| 1. Bertotti, 2018 | 17 | 18+ | Not reported. | Not reported. | Not reported. | Mild to moderate mental health problems. |
| 1. Bitter, 2017 | 263 | 18+ | 50.06 | Not reported. | 45% | Serious mental illness. |
| 1. Bjorkman, 2000 | 183 | 20-77 | 41 | Not reported. | 49% | Serious and/or enduring mental health problems. |
| 1. Bradshaw, 1998 | 9 | 24-63 | 39 | 100% White British | 44% | Long-term mental illness. |
| 1. Bragg, 2014 | 287 | 14-78 | 35.7 | 92.6% White British, 2.1% White Other, 0.4% Asian Indian, 0.4% Asian Bangladeshi, 0.7% Black Caribbean | 30.5% | All mental health problems. |
| 1. Calsyn, 1998 | 165 | None | 34.21 | Study 1: 47.5% caucasian,52% were African-American and .5% other minorities  Study 2: 45% Caucasian; 54.5% African-American | 42% | Major AXIS-I disorder and DSM-IIIR AXIS I diagnoses. |
| 1. Castelein, 2008 | 106 | Not reported, | 38.6 | Not reported. | 34.5% | Schizophrenia or a related psychotic disorder. |
| 1. Chang, 2016 | 156 | 15-25 | 22.9 | Not reported. | 48.1% | Psychosis. |
| 1. Chowdhary, 2016 | 271 | 17+ | Not reported. | 100% Indian. | Not reported. | Moderate to severe depression. |
| 1. Darongkamas, 2011 | 10 | 24-50 | 33 | Not reported. | 0% | All mental health problems. |
| 1. Felton, 2009 | 22 | Not reported. | Not reported. | Not reported. | Not reported. | All mental health problems. |
| 1. Fieldhouse, 2003 | 9 | 24-61 | 46 | 5 White, 1 African-Caribbean, 1 Thai, 1 Kurdish and 1 Indian. | 66.6% | Serious mental illness. |
| 1. FitzGerald, 2011 | 43 | 21-59 | 36.19 | White British (86%), British Afro-Caribbean (7%) and British Pakistani-Asian (7%). | 16% | Serious mental illness. |
| 1. Fowler, 2018 | 154 | 16-35 | 24.5 | 73% White British | 24.5% | Non-affective psychosis. |
| 1. Friedrich, 2018 | 30 | 20-56 | 34 | White British: 7  Black Caribbean: 6  Black African: 5  Mixed race: 5  Asian Indian: 3  Asian Pakistani: 3 | 7% | Psychotic and affective disorders. |
| 1. Garety, 2006 | 144 | 16 - 40 | 26 | Intervention group 38% white, control group 25% white. More than half of participants were from a minority ethnic, predominantly of African or Caribbean parentage | 35% | Psychosis and schizophrenia. |
| 1. Gater, 2010 | 123 | Not reported. | 41.7 | British Pakistani. First-gen Pakistani - antidepressant group: 34 (81%), social intervention group: 32 (82%), combined treatment group 37 (88%) | 100% | Depression. |
| 1. Hacking, 2008 | 149 | 16-64 | Not reported. | Not reported. | 33% | Long-term mental health problems. |
| 1. Hanlon, 2019 | 12 | 34-64 | Not reported. | Not reported. | 50% | All mental health problems. |
| 1. Hanly, 2020 | 14 | 19-52 | 30 | Not reported. | 82.9% | Eating disorders. |
| 1. Haslam, 2016 | 156 | Not reported. | 20.6 | Not reported. | 70.7% | All mental health problems. |
| 1. Haslam, 2019 | 120 | 18+ | 31.06 | Not reported. | 64% | Adults presenting with psychological distress in association with loneliness. |
| 1. Hassan, 2021. | 18 | 34-65 | Not reported. | White British n=14/18; no further data reported | 66% | Not reported. This study explored the experiences of secondary mental  health care service users use of the Life Rooms |
| 1. Howarth, 2018 | 47 | 35-68 | 53.2 | Not reported. | Not reported. | Participants with a range of mental health problems who were at different stages of recovery. |
| 1. Johnson,, 2018 | 441 | Not reported. | 40 | White: Intervention (65%) control (65%)  Black (UK, African, Caribbean, and Other) intervention (20%) control (19%)  Asian (UK, south Asian, Chinese, and Other) intervention (6%) control (6%)  Other intervention (9%) control (11%) | 60% | All mental health problems. |
| 1. Kaltman, 2016 | 28 | Not reported. | 48 | Latina immigrants to the US. The greatest proportion of women was from Central America (61%, n=17); the rest were from South America. | 100% | PTSD and/or depression. |
| 1. Lund, 2019 | 19 | 26-69 | 45 | Not reported | 73.7% | All mental health problems. |
| 1. Mak, 2016 | 118 | Not reported. | 42.9 | 100% Chinese | 58% | All mental health problems. |
| 1. Margrove,2013 | 95 | 20-65+ | Not reported. | White British control 28 Intervention 24 Transfer 18.  White Irish control 1 Intervention 1 transfer 0  Black African Control 1 Intervention 0 Transfer 0  Mixed or other. Control 1 intervention 0 transfer 0  Not provided control 1 intervention 1 transfer 1 | Not reported. | All mental health problems. |
| 1. Mathias, 2019 | 112 | 12-24 | 18.9 | Not reported | Not reported. | Self-reported mental health problems. |
| 1. Mazzi, 2018 | 48 | 18+ | Not reported. | Not reported | 29.5% | Schizophrenia and psychosis. |
| 1. O'Brien, 2011 | Not reported. | 22-60 | Not reported. | 45% White British, 20% Black or Black British African, 15% Asian or Asian British, 10% White European, 10% Black or Black British Caribbean | 40% | All mental health problems. |
| 1. O’Connell, 2020 | 137 | Not reported | 37.9 | African-American: 58%  White: 20%  Other: 12% | 34% | Psychosis. |
| 1. Petryshen, 2001 | 36 | 18+ | 43.2 | Not reported. | 61.1% | Variety of mental health problems and  psychiatric diagnoses. |
| 1. Rivera, 2007 | 255 | Not reported. | 38.3 | Caucasian: 58 (29%) African American 35 (17%) Hispanic 62 (31%) Other: 48 (24%) | 100% | Axis 1 disorders. |
| 1. Segal & Holschuh, 1991 | 234 | 18+ | Not reported. | Not reported. | 47% | Serious mental disabilities (excluding persons with developmental disabilities); |
| 1. Sexton, 1992 | 16 | 34-75 | 59.3 | Not reported. | 89% | Long-term mental health difficulties |
| 1. Sheridan, 2015 | 118 | Not reported. | 51 | Not reported. | 52.3% | Serious mental illness |
| 1. Sheridan, 2018 | 70 | 21-74 | 48.77 | Not reported. | 50% | Enduring mental illness |
| 1. Snethen, 2012 | 8 | 24-57 | 39.88 | 1 African American, 7 White | 37.5% | Schizophrenia spectrum disorders. |
| 1. Suto, 2021 | 23 | 32-67 | 53.11 | Not reported. | 35% | All mental health problems (self-reported). |
| 1. Tarbet, 1985 | 10 | 22-56 | Not reported. | Not reported. | 60% | Former psychiatric patients, all of whom had been hospitalized for psychiatric treatment at least once. |
| 1. Tempier, 2012 | 144 | 16-40 | 25.85 | White Specialised Care (SC) (n=24) 42% Standard care (ST) (n=12) 24%  Black SC (n=24) 42% ST (n=29) 58%  Other SC (n=9) 16% ST (n=9) 18% | 34.5% | First episode of psychosis. |
| 1. Terzian, 2013 | 357 | 18-45 | Not Reported. | Not reported. | 31.3% | Schizophrenia spectrum |
| 1. Thorup, 2006 | 547 | 18-45 | Not Reported. | Not reported. | 40.9% | Schizophrenia and psychosis. |
| 1. van der Venter, 2014 | 44 | 27–73 | 43 | Quant: 29 White British, 9 BME, 6 not reported.  Qual”: White British, Asian, White British, Black British, White British, White British. | 66% | People with mild-to-moderate mental health problems. |
| 1. Varga, 2018 | 75 | Not reported | 39 | Not reported. | 49.3%. | Schizophrenia. |
| 1. Webber, 2019 | 155 | 16-87 | 41.7 | Most were of White British ethnicity (n 1⁄4 125, 80.6%); others were Asian (n 1⁄4 15, 9.7%), Black (n 1⁄4 5, 3.1%), or of other ethnic origin (n 1⁄4 10, 6.2%). | 45% | All mental health problems. |
| 1. Webber, 2021. | 159 | Not reported. | 41.4 | 100% White. | 68.5% | All mental health problems. |

**Table S3: RCT Outcomes (n=17)**

| **STUDY ID:** | **Social network measure** | **Social network measure data** | **Other relevant social network data.** | **Other outcomes** |
| --- | --- | --- | --- | --- |
| Ammerman, 2013 | Social Network Index. Network Size Subscale; Network Diversity Subscale; Network Embeddedness Subscale. | No group differences were found in size of and involvement with social networks. For each scale, both groups either remained stable over time or showed increases over time. | Those receiving IH-CBT reported increased social support over time relative to those in the SHV condition. Effect sizes were modest at post-treatment (p < 0.001, ES=0.38) but increased at follow-up (ES=0.65).  Improvements were seen in affiliative and belonginess aspects of social support, in contrast to tangible support which was statistically non-significant. Findings were not moderated by clinical features of depression or home visiting parameters. | Subjects receiving IH-CBT reported decreased psychological distress at post- treatment (p < 0.001, ES=0.77) and follow-up (ES=0.73). Examination of types of psychological distress indicated broad improvements at both time points. |
| Bitter et al, 2017 | None | None | Social functioning was measured  using the Social Functioning Scale (SFS).  Social functioning. No significant differences noted post intervention. |  |
| Calsyn, 1998 | Social network size; Arizona Social Support Interview Schedule with additional items; Personality and Social Network Adjustment Scale | **Study 1:** The be- tween group differences in the size of the natural support networks approached, but did not reach, statistical significance, F (2, 109) 4 2.67, p , .07. However, there was a significant difference between treatment groups in terms of the size of the professional network, F (2, 109) 4 6.84, p , .002. Post hoc analyses indicated that clients in the ACT condition had significantly more professionals in their networks than clients in the other conditions.  **Study 2:** There was a significant treatment group effect on the network size variables, Wilks Lamda (6, 252) 4 .84, p , .0001. Although there was no significant difference between treatment groups in terms of the size of the natural support network, there was a significant difference in the size of the professional network, F (2, 126) 4 10.47, p , .0001. | **Study 1:** There was no significant difference between treatment groups on the type of social support received, F (10, 210) 4 .77, p , .66, 4 .93. MANCOVA was also used to analyze the qualitative dimensions (network satisfaction, interpersonal adjustment, and alienation). There was no significant between group differences on the qualitative variables, F (6, 216) 4 .43, p , .86, 4 .98.  **Study 2:** There were no significant treatment group differences on the emotional, advice, recreational and conflict dimensions. However, there was a significant treatment group difference on the material assistance dimension, F (2, 122) 4 7.20, p , .001.  There were no significant effects of treatment group, time, or treatment group by time on the qualitative measures of social relation- ships. There was a significant effect of treatment on the interviewers’ ratings of the adequacy of the social network, Wilks Lambda (6, 232) 4 .73, p , .0001. | None reported. |
| Castelein, 2008 | Self-developed list [Personal Network Questionnaire (PNQ)] asking for information on the frequency of contacts with named family, friends, and members of the peer support group. | Participants had a significant increase in contact with peers outside of the sessions (P = 0.03) and on esteem support (i.e. asked for advice, received a compliment, asked for help; P = 0.02) in comparison with the WLC condition (56% improvement vs 31% improvement). The positive effect on peer contact did not generalize to other relationships; for instance, contact with family and friends. | More negative symptoms at baseline (P = 0.02) and more distress caused by these symptoms (P = 0.05) predicted improved psychological health, but not on social relations (P = 0.01). More distress caused by positive symptoms (P = 0.05) and a longer duration of illness (P = 0.06) predicted improved social relations. Those with higher distress from negative symptoms had significantly less chance of improving on social relations (P = 0.01). | **Symptomology:** the participants in the experimental condition had statistically significant, fewer negative symptoms (P = 0.02) and less distress from these symptoms (P = 0.04) in comparison with the participants in the control condition. In addition, no between-condition differences were found in hospitalization rates (P = 0.28) during the intervention.  **Self-efficacy, self- esteem, and self-reported quality of life:** no between-condition differences were found, but participants in both conditions improved over the study period.  The high attender group significantly improved on social support, self-efficacy, and quality of life compared with the low attender group. |
| Chang, 2016 | None | None | Psychosocial functioning was measured by Social and Occupational Functioning Assessment Scale (SOFAS) and Role Functioning Scale.  Extended early intervention group displayed significantly better outcomes than standard care group in social functioning at the end of 12-month follow-up. | **Symptoms:** Extended early intervention group displayed significantly better outcomes than standard group in negative and depressive symptoms at the end of 12-month follow-up  Female gender, lower degrees of premorbid schizoid-schizotypal traits, extended EI treatment condition, lower levels of positive symptoms at intake, and better baseline functioning independently predicted FR |
| Fowler et al, 2018 | None | None | Social recovery therapy plus early intervention services was associated with a large and clinically important increase in structured activity of 8·1 h (95% CI 2·5–13·7; p=0·0050) compared with early intervention services alone. |  |
| Gater, 2010 | None | None | A social functioning measure specifically created for British Pakistani women. There was a greater increase in social functioning in the social intervention group and the combined treatment group than in the antidepressant group at both 3 and 9 months, but these were significant only at 3 months. | **Symptoms:**  Hamilton Rating Scale for Depression (HRSD) – no significant difference  Depression remittance – no significant difference. |
| Garety, 2006 | None | None | None. | **Symptoms:** The Positive and Negative Symptoms Scale – trend towards the intervention group but not significant after adjusting for differences in baseline variables.  Calgary depression rating scale – no significant differences between groups.  **Functioning:** The Global Assessment of Function –  Significant differences favouring the intervention group.  **Insight:** 18 months: The Scale for the Assessment of Insight – no significant differences.  **Treatment adherence, user satisfaction and self-rated quality of life** -significant differences favouring the intervention group. |
| Haslam, 2019 | None | None | Roberts UCLA Loneliness Scale: Both the main effect of time, B = - .35, p < .028, and the Time x Condition interaction were significant, B = - .74, p < .001. Loneliness decreased significantly in the intervention group, t(97.0) 7.27, p < .001, but not in TAU, t(90.8) = 2.23, p = .098.  People’s sense of belonging to multiple groups was assessed using a standard four-item multiple group membership scale  Both the main effect of time, B = .37, p < .010, and the Time x Condition interaction were significant, B = .52, p = .009. Group memberships increased significantly in the intervention group, t(95.8) = 6.57, p < .001, and also increased significantly in TAU, t(91.1) = 2.64, p = .036. However, the results for depression and GP visits were not significant. | **Depression and number of GP visits:** No significant differences between group.  **Social anxiety:** social anxiety decreased significantly for those in the intervention group, t(85.9) = -4.22, p < .001, but not in the TAU condition, t(83) = .25, p = .993. |
| Johnson et al, 2018 | Lubben Social Network Scale | At 4 and 18 months, there was little evidence of any effect; the difference in social networks favoured the intervention but it was not statistically significant | None. | **Service use:** Readmission to acute care within 1 year was significantly lower in the intervention group than in the control group (p=0.0438). Time to readmission was significantly longer in the intervention than in the control (p=0.0291). However, the number of days in acute care was not significantly different. Participants in the intervention group had fewer days in acute care than did participants in the control group, but the difference was not significant.  **Satisfaction:** At 4 months of follow-up, overall satisfaction with mental health-care received was statistically greater in the intervention group than in the control group ( p<0·0001).  **Recovery:** There was also a significant difference in self-rated recovery favouring the intervention, but the difference was not significant in sensitivity analysis with adjustment for predictors of missingness. |
| O’Connell, 2020 | None. | None. | The social functioning scale (SFS): A significant group by time interaction was found for social functioning (F(4,279) 3.13, p = 0.02). Participants in the intervention group (t(279) = 1.76, p = 0.08, b = 0.27) and Engage (t(279) ¼ 3.47, p = 0.001, b = 0.53) conditions had significantly greater increase in social functioning from baseline to nine months than those in TAU.  Relatedness: A significant group by time interaction was found for relatedness as measured by the DES (F(4,251) = 2.47, p = 0.05). Participants in the Engage condition had a greater increase in relatedness than those receiving TAU from baseline to three months (t(251) = 2.63, p = 0.009, b = 5.56). | **Symptoms:** The Positive and Negative Syndrome Scale (PANSS): Significant differences favouring the intervention group for positive symptom (F(4,262)=3.16, p=0.02) and negative symptoms (F(4,262)=2.44, p=0.048).  **Service use:** A significant group main effect was found for inpatient readmission (F(2,133) = 3.50, p = 0.03). Participants in intervention groups had significantly fewer readmissions from baseline to six months and from baseline to 12 months than those in TAU. |
| Rivera, 2007 | Pattison Network Inventory (31,32). This interview assessed social network size, total number of social contacts, degree of reciprocity of relationships, density of the social network, and the number of times the client was helped or had helped others in his or her network. | Clients receiving peer-assisted care showed a significant increase in the number of contacts from baseline to 12 months. Follow-up analyses revealed that this effect was due to increased contact with peer assistants and professional staff, not with family and outside friends. There were also significant improvements for all conditions in several other network measures as indicated by reliable main effects of time: total number of others involved in social activities, total number of others who helped client, total number of others helped by client, and network density.  Peer-assisted care showed the greatest increase in self-reported social contacts with consumer and professional staff. Peer assistants provided planned activities and regularly scheduled home visits to enhance the social network. These increases did not extend to kin social contacts, |  | Data indicate that although the three programs had distinct patterns of services, they yielded the same general pattern of improvement over time on a variety of measures: symptoms, health care satisfaction, and various ratings of the quality of life. Clients in the three programs also showed similar but small changes in measures of social network behavior. No one program emerged as categorically superior to the others. |
| Sheridan, 2015 | Practitioner Assessment of Network Type | At baseline, approximately 54% of the partnered and 57% of the un-partnered group were living in social networks with relatively limited contact with friends or neighbours and approximately 40% of both groups in the two most socially vulnerable networks. The main change over the course of the study was the extent to which participants reported having contact with friends on a weekly basis. However, the proportion of respondents in both groups who had no friends remained relatively unchanged.  Although there was variability in the extent to which the social networks of the control and intervention groups changed through- out intervention, there was a decline in the proportion of participants in the two most vulnerable networks. |  | **Beck depression Inventory:** No significant difference between groups..  **Loneliness:** Social and family loneliness decreased significantly over time for both groups. Romantic loneliness scores also decreased, the decrease was not statistically significant.  **Rosenberg’s self-esteem measure:** Both groups demonstrated good levels of self-esteem and levels remained consistent for both groups throughout the study.  **Social functioning:** involvement in social recreational activities increased significantly over time for both groups and there was a slightly higher level of recreational involvement in the partnered group; however, the difference between both groups was not statistically significant. |
| Tempier et al, 2012 | Social network size; functional adequacy of social support as measured by the gap between ideal and perceived levels of support. | The mean network size was not statistically different between groups at 6 months, although there was a trend toward bigger networks among specialized care patients. When members of the patient’s network were classified by role, for example, parent or sibling, participants in the specialized care group tended to report more responses for each role, suggesting that their net- works were denser.  Patients in specialized care reported having a higher number of significant others in their social networks than did standard care patients (2.40±1.20 and 1.71±1.06, respectively; p= .01).  At 18 months, the intervention group had a larger network than the standard care group. | A comparison of emotional and practical support subscales of the SOS showed no difference between groups in ideal and perceived levels of support. For all subscales, the perceived level of support was less than the ideal level. | **Symptoms:** No significant differences were found in either clinical outcome measures between the two treatment groups at six- month follow-up.  At 18 month follow-up the intervention group scored significantly better on the negative PANSS, total PANSS, and GAF. |
| Terzian et al, 2013 | Social network size: Number, frequency, importance, or closeness of relationships | A social network improvement was observed at year 1 in 25% of the patients allocated to routine treatment and in 39.9% of those allocated to the experimental arm (OR 2.0, 95% CI 1.3 to 3.1; adjusted OR 2.4, 95% CI 1.4 to 3.9).  The difference remained statistically significant at year 2. | Participants attributed higher value to arms length relationships rather than friendships or confiding relationships.  Results suggest that improving social networking produces beneficial effects in patients with a better clinical prognosis. E.g. a good clinical prognosis might anticipate a good response in terms of social network improvement. This is not the case for the other outcomes, as the experimental treatment appears to be effective by and large regardless of improvements or worsening of work or activities of daily living. | No significant differences emerged for any of the other end points (Brief Psychiatric Rating Scale/Global Assessment of Functioning/Self-care/Activities of daily living/Hospitalisation/Work). However, patients with 1 or more other areas of improvement at year 1 and 2 showed a statistically significant social network improvement. |
| Thorup, 2006 | Social network size: Number of contacts with family and friends | The type of treatment did not affect the social network size after 2 years of intervention, since the distribution was not significantly different for ST and IT. At 2-year follow-up, IT patients had on average 4.3 contacts with family members in the previous month, while ST patients had on average 4.7 contacts (P = 0.28). The average number of contacts with friends during previous month was 3.8 for both IT and ST patients. | The final models included both age and network size at entry, but while the final model for family-network size included male gender and disorganised dimension, the friends-network size model included negative symptoms and A-level status as the significant variables. The univariate models show that dependence syndrome leads to less family contact, while not having completed high school and poor academic premorbid functioning do not. The geographical variable ‘site’ only has significant impact on number of friends. | Increased social network size at follow-up was closely related to younger age, being female, having completed A-levels, less negative symptoms, larger network size at entry. |
| Varga, 2018 | None | None | None | **Mental health symptoms:** All the patients remained in remission during the 6 months. No significant symptom worsening, relapse or hospitalization were detected during the observation.  **General functioning:** In addition, compared to baseline GAF scores increased significantly after 6 months both in the Commuity Club group (p < 0.001) and in the Case Management group (p < 0.001. |

**Table S4: Other quantitative outcomes (n=12)**

| **STUDY ID:** | **Social network measure** | **Social network measure data** | **Other relevant social network data.** | **Other outcomes** |
| --- | --- | --- | --- | --- |
| Aggar, 2021. | None. | None. | **Loneliness:** There were no significant differences in self-rated loneliness from pre- to post-intervention (MB = 6.4(2.29), MF = 6.1(2.17), t(10) = 0.412, p = 0.689.  **Social participation:** There were no significant differences in social participation from pre- to postintervention (MB = 2.7(1.32), MF = 2.9(0.94), t(10) = 0.232, p = 0.821). | **General Health:** Self-report health status improved significantly from pre to post-intervention (MB = 59.1(18.68), MF = 71.7(14.82), t(9) = 2.964, p < 0.05).  **Quality of life:** Self-report overall health satisfaction (MB = 2.4(1.00), MF = 3.3(0.97), t(10) = 3.194, p < 0.05), physical QoL (MB = 11.6(1.78), MF = 13(3.620), t(10) = 2.451, p < 0.05), and psychological QoL (MB = 10.3(2.45), MF = 12.4(2.96), t(10) = 2.909, p < 0.05) improved significantly from pre- to post-intervention. However, overall QoL (MB = 3.3(0.45), MF = 3.5(0.90), t(10) = 1.305, p = 0.221), social relationships (MB = 12.3(3.63), MF = 12.9(4.24), t(10) = 1.041, p = 0.322), and environment QoL (MB = 14.4(2.67), MF = 14.8(3.21), t(10) = 1.44, p = 0.18) did not improve significantly. |
| Bailey et al, 2020 | None. | None. | **Social connectedness:** Social Connectedness Scale-Revised (SCS-R)  8 week follow up score 27.9 (SD8.2) non-significant (p=0.062) improvement. | None |
| Becker, 1998 | The social network scale (Dunn, et al 1990; Leff et al., 1990) | Nunhead (intensive care): 14.3, p<0.01 SIGNIFICANT IMPROVEMENT. This applied to overlapping category of confidents too (friends, relatives, others).  Norwood (standard care): 17.8, P=0.36 NON-SIGNIFICANT DIFFERENCE. | Network increases were significantly higher for women vs men (p=0.01). It was higher for married vs single participants in total network size (p=0.01) and others (non-friends) p=0.03. | None. |
| Bjorkman, 2000 | None. | None. | More brokerage interventions, intervention planning and ADL skills interventions predicted a lower level of needs at follow-up.  More assessment interventions and more work time spent on indirect contacts on behalf of the client predicted a lower level of symptoms at follow-up, while advocacy interventions predicted a higher level of symptoms.  More interventions in the life areas of work and education predicted a better psychosocial functioning at follow-up, while interventions in the area of housing predicted a worse psychosocial function.  Interventions in the life area of finances predicted a better global functioning according to the GAF at follow-up. | None. |
| FitzGerald, 2011 | None | None | None | **Occupational functioning:** Model of Human Occupation Screening Tool (MOHOST). Significant improvements in the intervention group following intervention. This difference continued to be evidenced in four of the six subscales used: motivation for occupation, patterns of occupation, motor skills and environment all showing significant differences. |
| Hacking, 2008 | The Inclusion Web. | Mean data: There was a statistically significant increase in mean scores for both people and places and therefore also in the total measure, clockspread.  Places: Changes were highly statistically significant in Volunteering (163% increase in baseline score), Education (90% increase), Faith (83% increase), Sport (63% increase) and, to a lesser extent, Family and Neighbourhood (21% increase).  People: The largest and most significant increases appeared in Sport (205% increase in baseline scores), Volunteering (125% increase), Education (99% increase) and Family and Neighbourhood (13% increase). Employment, Arts and Faith domains fell just short of significance levels. | None. | There were no significant differences for Employment and Services. |
| Haslam, 2016 | None | None | **Social connectedness:** Significant improvements in loneliness p<0.001 and social functioning p=0.39.. | There was significant improvement in mental health (depression p=0.46, anxiety p<0.001, and stress p<0.001), wellbeing (life satisfaction, p=0.11 and self-esteem p=0.40). Benefits were underpinned by participants’ increased identification with the intervention group and with other groups. |
| Mazzi et al, 2018 | None. | None. |  | **General functioning:** Results showed a higher level of functioning in those subjects who had completed the intervention [intervention mean=67.4 (SD=13.4) vs wait list mean= 52.0 (SD = 17.5), p = 0.0006.  **Activity levels:** Intervention reported higher scores at the Measure of Activity in Psychosis [SP=mean 2.8 (SD=0.9) vs WL mean=2.2 (SD=0.9), p = 0.0129].  **Symptomology:** The HoNOS/MHCT showed a significant difference between the two groups, with regards to the “So- cial problems” subscale, which presented more favourable scores in intervention subjects.  **Self-esteem/internalised stigma/quality of life:** favoured the control group.  **Service use:** No significant differences between groups.  Analysis revealed the more social activities participants engaged with the better they performed on follow-up outcome measures. |
| Petryshen, 2001 | None | None | **Loneliness:** Significant improvements: [t (35) = 3.67, p < .001].  **Social functioning:** significant improvements: [t (35) = -5.33,p < .001]  **Satisfaction with social relationships:** significant improvements: [t (35) = -5.33,p < .001]  **Satisfaction with Leisure**: significant improvements (t (35) = -2.50, < .05] | **Self-esteem:** The Rosenberg Self-esteem Scale. Significant improvements in self-esteem: [t (35) = 2.65,p < .05]  **Quality of life:** Significant improvements in general life satisfaction at the one year follow-up than at intake [t (35) = -3.58, p < .001.  Greater levels of participation were associated with greater benefits.  Married participants improved more than those who were single or divorced or separated. Those living with a spouse/partner improved more than those who lived alone or in supportive housing. |
| Segal & Holschuh, 1991 | Pattison Psychosocial Kinship Inventory (PKI). The PKI elicits information on network size and composition and characterizes the type of support, as emotional or instrumental, and its directionality, as given or received. | Sheltered care had a positive effect on the residents’ development of social networks. For each additional episode in a sheltered care facility from 1973 through follow-up, residents were 23 percent more likely to have an emotionally supportive relationship, 22 percent more likely to have one in which they gave emotional support, and 29 percent more likely to have an instrumentally supportive relationship. |  |  |
| Webber, 2019 | None. | None. | Social capital: Resource Generator-UK (RG-UK, Webber & Huxley, 2007).  Participants exposed to practice with high fidelity to the Connecting People Model had significantly higher access to social capital (p 1⁄4 .03, partial Z2 1⁄4 .05) and perceived social inclusion (p 1⁄4 .01, partial Z2 1⁄4 .07). | **Health economics** - financial benefits of intervention: The overall mean cost of services people used in the high- fidelity CPI group reduced from £2,775 at pretest to £1,807 at posttest. Mean costs in the low-/moderate-fidelity CPI group were higher but similarly reduced over time from £8,203 at pretest to £4,092 at posttest. The difference at posttest was statistically significant (£1,331, 95% confidence interval [CI] 1⁄4 [£69, £2,593]) |
| Webber, 2021 | None. | None. | The primary outcome was access to social capital, as measured by the RG-UK (Webber and Huxley, 2007). No significant improvements at follow-up | Goal Attainment Scaling (GAS)/Recovery/Wellbeing/Quality of life/Health care use: No significant differences at follow-up. |

**Table S5: Mixed methods outcomes (n=12)**

Key to coding frame for qualitative and mixed methods studies:

**Red: Impact of relationship with facilitators** – ~~may need to be split into~~ including Bridging the Gap and **Skill of the Worker**; **peer or professional; training/supervision**; modelling behaviour; trust; creating bonds; **genuine**; understanding; boundaries; person-centred approach; recommend to others; feedback loop; **level of managerial buy-in**; building plans; non-clinical v clinical

**Beige: My Choice, My Pace, My Life** - **self-selected interests that are motivating to the individual;** **going at own pace**; an understanding of needing to be flexible so others have their choice too; freedom; sense of fun/enjoyment; being given 'permission'; **readiness to change**; managing expectations; awareness/assertion of rights; empowering; autonomy

**Blue: Social Building Blocks** – **internal and external social resources and skills, rebuilding or newly acquired**; reference to lack of such skills or maladaptive coping prior to intervention (e.g. avoidance, addiction); building resilience; dealing with endings; **translatable skills/environments**; **re-evaluation of self; self as an asset**; first-hand experience allays doubts/fears; courage

**Burgundy: Purpose in Life** – includes **building routine, contributing to community**, **something to get up for, get out of the house for**, able to achieve flow with; reciprocity; **a focus away from MH**; **intrinsic pleasure of the activity; importance of being present**

**Purple: Making Connections** - **shared interests (rather than) shared diagnoses**, **natural relationships v. ‘intentional friendships’**; connections in community highly valued; **stress of making new connections**; collective efficacy; kindness

**Pink: Barriers to accessibility/availability and sustainability of activities**: Includes health, transport, funding issues, personal finance, gender issues, family and other support, other commitments (e.g. work, caring responsibilities); own fears and anxieties; frontline staff reluctance

**Orange: Other Life Change outcomes during/after intervention -** (e.g. wellbeing, employment, education, housing, medication, recovery from addiction)

**Green: Safe and positive Space -** safety, possibly nature, a conduit to social inclusion and better health; non-judgemental

**Grey: Maintenance of activities/networks; encouragement of this; evidence of reach beyond programme; stepping stone**

| **STUDY ID:** | **Social network measure** | **Social network measure data** | **Other relevant social network data.** | **Other outcomes** |
| --- | --- | --- | --- | --- |
| Bertotti et al, 2018 | None | None | **Component 1 - GP Referral process:** Outcomes: Referral to social prescribing coordinator **Component 2 - Interaction with social prescribing coordinator:** Outcomes: (i) Increased trust, hope and self-esteem from interaction (ii) Facilitated access to further support from community/statutory organisations **Component 3 - Interaction with community/statutory organisations:** Outcomes: (i) Improved health and wellbeing (ii) Improved social interaction between users  *‘Last summer was the best one I’ve had since my surgery because I’ve got somewhere to go….. It’s better to sit with company sometimes than sit by yourself.’ (service user) (E) ‘Best thing has been meeting new people and making friends. My mobile full up with names and numbers of friends before it was just family and doctor’s number.’ (service user) (E)*  **OUTCOMES** Increased satisfaction with meeting social prescriber. Increased activation. Reduced loneliness. Increased self-esteem Increased social networks Reduction in health care resource use. Improved mental wellbeing. Improved quality of life | Social prescribing improves wellbeing outcomes for patients suffering from isolation, mild mental healthproblems by providing a support mechanism which enable each individual to consider a set of alternative actions and thus embark in changing their current health. |
| Howarth et al, 2018 | Recovery Star | The repeated recovery star scores indicated a positive trend in the social networks, and an overall improvement of 45% was recorded. | Collectively, the recovery star data indicated an improvement in mental health social networks and relationships.  The qualitative data support these [social network quant] findings, and being connected and integrating with others was viewed as a positive outcome for participants who were interviewed.  The recovery journey was influenced by the volunteers’ ability to reengage in social activity, which is often difficult for people with mental health difficulties. Attending the MHRP and using TH approaches that involved shared activities helped individuals to reconnect: *What worked for me? Getting involved really. Helping others out. I seem to know a bit myself, like what I could show other people how they run it. (Exit interviewee 6)* Participation in TH also enhanced the development of new skills as a result of engaging with nature-based activities such as building raised beds, sowing seeds and creating planters for sale. These activities enabled participants to re-engage in a social life, in its broadest sense | The recovery star data indicated whether the participants were able to manage their mental health. The baseline score revealed that 27% of participants had recorded that they were managing their mental health, and 26% reported that they were learning how to manage their mental health. However, just under half (47%) indicated that they were actively seeking help to manage their mental health. The data suggest that the MHRP attracted people with a range of mental health problems who were at different stages  of managing their mental health (see Figure 1) (ADF - data not clear from graph so couldn't transcribe it).  The second recovery star score highlighted that there was some improvement in the  mental health scores for 35% of the participants and 45% recorded similar scores to the baseline. Overall, the recovery star data suggest that the MHRP had enabled participants to move from seeking help to learning to manage their mental health.  **‘the recovery journey’** (Transferable skills; Skills to move on and get a job; Coming out of the house for the first time; Re-engaging in a social life) (E)  …and the qualitative data highlighted that participants felt they were developing skills that would assist them in gaining employability. There was also a sense of gaining confidence, which is summed up by one of the participants: *My confidence is ecstatic. The more I work, the more my confidence grows. (Exit interviewee 2)* Some of the participants were retired due to medical reasons, yet there were four participants who had gained meaningful employment as a result of attending the MHRP. As such, reengaging with others indicated a growth in confidence which enabled participants to reengage outside of the MHRP context. |
| Bragg, 2014 | None | None | A paired samples t test showed a statistically significant increase in participant social engagement and support scores [t(66) =-3.895, p<.OOl; 112=.36 - large effect size] from the start (M=13.64, 5D=2.47) to the end of Ecominds programme (M=14.72, 5D=2.29) representing an improvement in social engagement of 1.12. The percentage change between baseline and endpoint ranged from a decrease of 22% to an increase of 89% with the mean increase being 10%.  The single-item measure on community belonging was asked at the beginning and at the end of the green care programmes. Mean and median scores were similar for both time points: baseline M=2.60, 50=0.83, Mdn=3 and at endpoint M=2.50, 50=0.68, Mdn=3; and no significant differences were found when tested with a Wilcoxon Signed ranks test (p=.S88).  29% of participants experienced increase in community belonging. 81% of participants increased frequency of involvement in community activities by endpoint.  When participants were asked how frequently in the last year they had helped with or attended activities organised in their local area, at the start of the programme 42.6% of participants reported that they attended at least once a month and at the end of the programme this had risen to 69.7%. The majority (81%) showed an increase in the frequency of getting involved in other community activities after involvement with a green care programme. | "Sense of satisfaction upon completion of this long project (we've been working on this wall for many weeks)"(E)  "Working in wildlife area, clearing weeds from plot, picking vegetables, hoeing, drink and cake and friends"(E)  Calming and therapeutic activity(E) |
| Snethen et al, 2012 | None | None | The average number of activities based on all end of day DRM reports were M = 3.8 (standard deviation (sd) = 0.95), M = 4.7 (sd = 0.57) and M = 4.3 (sd = 1.1) for DRM collection during weeks 1, 4 and 10, respectively.  Participants who completed the exit interview identified three primary outcomes from the intervention:  (1) community involvement: **Activities means that you’re not locked up, you know?** When the door is out there and you can’t get out, you can’t do nothing. That’s a reminder that I can get out and do stuff, not locked behind the door.(E) (2) planning: “I’ve been taking it as it comes, but I’ve been wanting to get a set routine,” (E) “[I’d like] more structured activities.”(E) I just do the best I can do but I still don’t know. I was hesitant about making plans because I don’t know [how] I’m going to feel. And then like with the yoga and doing it on my own, I could[n’t] do it on the days that I was laying in bed on the same day I said I was going to do it. But I was able to do it on a day that I felt half way decent. (E) When asked about understanding the bus schedule, Arnie stated, “At the very beginning, there was anxiety big time… It’s over with now.” Through participation in the intervention, Arnie was able to overcome his anxiety about the bus schedule and learn to use the appropriate bus for different appointments, “just by you writing down things and stuff… I need to write down things myself, too. It all works hand in hand.” (E) | (3) coping: Jerome, who often had intrusive thoughts that made him feel depressed,was able to identify an activity to decrease stress and feel better about himself, “I learned I can go walk when I’m stressed out. Hop on the bus and go out to the trail and go down the trail.”  (E) Mary was able to list activities she could do both at home and in the community when she was feeling stressed or overwhelmed. This was important for her, as she had visited the emergency room twice in a 2-week period for psychiatric services, without consideration of other options. By the end of the intervention, she had identified the importance of finding activities she could do that helped her feel better when feeling depressed. (I) When discussing the activities she and the recreational therapist chose to participate in, Tracy identified the perceived participation benefits, “And they’re helpful. Like yoga is good for your body. And the pool is good to be in the sun for Vitamin D, or whatever…” (E) |
| Sexton, 1992 | None | None | Complexity of relationship between intervention/outcomes: The results showed that current attenders were using the group to extend their social contact: 81% either agreed or strongly agreed with the statements relating to intervention meeting that aim. Past attenders were much more unsure about this aim (40% were unsure) and only 50% could either agree or strongly agree with the relevant statements. 'meeting others' was rated highest as the most enjoyable aspect of the facility. BUT , 'other group members' was rated highest on the question 'What do you least enjoy about the group?'. | **Personal growth:** One past attender commented, 'I feel that I have become a person in my own right.'   **Provision of support:** 'The support I received from the group led to increased confidence to apply for a job at which I'm still employed (nearly a year)".  **Increased engagement in activity.**  The facility has also been effective in reducing the number of home visits made to long-term clients by team key workers (aim 6). Of current attenders, 68% either agreed or strongly agreed with the statements relating to this aim (Fig.7). This should allow the team to be more cost effective as well as providing a more appropriate style of intervention. |
| Mak, 2016 | Participants’ social network size was measured by items adapted from the Chinese version of the Medical Outcomes Study Social Support Survey (MOS-SSS-C; Yu, Lee, & Woo, 2004). | PRE: 13.64 (12.57) with matched controls at 14.49 (14.18). POST: 15.36 (13.46) with matched controls at 13.97 (13.39). NO SIGNIFICANT CHANGE IN SOCIAL NETWORK WORK SIZE. | Significant improvement in perceived social support post-intervention (p = .02). The results indicated that the change in perceived social support was found between pre- and postassessments and WRAP participants felt they were more supported after the completion of the program. Such increase in sense of support was observed at postassessment and sustained at 3-month follow-up after the program. | No significant change in empowerment, hope, self stigma, symptom severity and recovery. |
| Kaltman, 2016 | None |  | Perceived social support:  PRE:  Mean (SD) - 49.46 (17.53)  POST:  50.82 (16.30). No significant difference.  I - **Levels of social support did not change from baseline to post-intervention, counter to expectations.** Despite this finding, there was some evidence in the semi-structured interview data that women experienced improvements in their perceived social support. Women highlighted their enthusiasm for being with others like themselves and having the opportunity to share with and learn from others (n=16). (E) “The days that we were all together were beautiful because everybody could say what they felt… We were sad at the beginning, but then everyone was happy, everyone wanted to participate and talk.” (I) Some of the participants (n=4) also talked about connecting with the women from their group beyond the formal group sessions. (E)“I have new friends. Perhaps I can contact them in the future and I can rely on someone to talk to and unload because I have so much inside and I don’t have my family here with me.” | Significant improvements in symptom levels for depression and PTSD. |
| van der Venter, 2014 | None | None | None | Well-being was measured using WEMWBS: a validated fourteen-item scale (range 14 –70).Baseline, interim and outcome WEMWBS scores were captured… Mean baseline: 38.2, mean outcome: 46.2, mean change (95% CI): 8.0 (4.8, 11.2), difference in mean change (95% CI): T-test, P , 0.0001. These are further broken down by gender and ethnicity as discussed.  I - All participants reported benefitting from AoR, irrespective of their measured change in WEMWBS  Mean well-being improved following participation in AoR and WEMWBS scores increased with each AoR session. Well-being improved more slowly for participants with low baseline scores; however, qualitative findings suggest these individuals may find arts participation helpful in managing emotions and preventing deterioration of well-being. Triangulation also showed those with little/no improvement in WEMWBS experienced stressful life events during their referral. |
| Margrove et al, 2013 | None. | None. | Significant improvement in social inclusion: A paired samples t-tests showed that there were improvements in SIS scores over time for the intervention group (t = -1.86, df = 25, p < .05) only.  Significant improvement in wellbeing: paired samples t-tests showed that there were improvements in well-being scores only for the intervention group (t = -3.06, df = 25, p < .01).  The social gains identified by participants included increased motivation to do arts and other activities, enhanced social skills and reduced social isolation: *I have gained experience and learnt to be quiet while I was painting… I learnt how to cope with being part of a team /  I got more out of it than I thought, I felt part of a group. (E- Male) [When I went home] I made phone calls, talked to people / For me it was mixing with other people, that was good, and seeing their work / Yes, socially we were encouraging each other / I have made some exceptionally nice friends. (E - focus group)* For some participants, a sense of expanding horizons, in terms of further opportunities, was further evidence of social gains: *The structure of having something to do for two hours. I have a bad back and I didn’t think I could do anything for two hours… I have realised now that I can… so now I think perhaps I can volunteer at the graphics place [a local enterprise]. (E -Male) I’m hoping to do voluntary work for the Salvation Army. Art will always be there but I can pick it up whenever I want now. The main thing is finding something suitable for myself and to help other people. (E -Male) I do other arts and I’ve been going round looking at homes and feel I can offer my support and my art as a volunteer. Bring it out in someone else. It’s opened my eyes to help other people. (E - Female)* | The majority of the service users reported enjoying the Open Arts course (96.2%), felt that their art skills had improved (96.1%), reported increased confidence (80.5%) and  reported improved motivation (88.4%).  Most also said that they felt more positive about things (73.1%) and that their relationships with other people had improved (88.5%).  All 19 participants identified improvements in mental wellbeing and social gains that they attributed to their course. The improvements in mental wellbeing described included increased confidence, relief from worries and a sense of achievement. An optional part of the Open Arts course is to try for a qualification. For participants who had completed their Open College Network accreditation, this had clearly contributed to their pride in their achievements: *I was really pleased with what I had achieved. I got positive comments… The best bit is doing the accreditation at the end, I felt pleased and proud of my work /  It keeps my mind occupied. I didn’t do too brilliantly but I gave it a go. (E - Male and female) I think it’s brilliant, it has done a lot of good being in a different world… I felt I was doing something useful with my life, creating something beautiful… I used my mind to produce something beautiful, it’s never too late to learn. (E- Male)* |
| Darongkamas, 2011 | None | None | Author interpretation and quotes in quotation marks: **Theme: Social inclusion** Participants made references to the social nature of participating in football throughout the interviews, all participants commenting on it at some point. Socialising within the team was a common theme, five participants stating that they ‘socialise a lot with other players’. Participant P6 and P7 both referred to the ‘camaraderie among players’ as an important part of their participation. Three comments were made about friendships with other players. P7 also reported that: ‘The friendship in the squad is very sincere and honest, it’s not false’. Participation in the football team also encouraged socialising outside the football club, three players saying that they ‘go out more’. P2 commented: ‘I wasn’t one for socialising before – I used to make excuses to stay in, but now I make the effort to go out’. P1 said: ‘It’s helped me get back in contact with friends from the past... It’s encouraged me to meet people’. P9 also reported benefitting from the social aspect of the club. | Overwhelmingly participants reported feeling positive. All participants said they enjoyed the football and felt comfortable in the team.  In relation to perceived changes resulting from the football, most players reported improvements to their mental health, attitudes about themselves and general well-being. The majority of participants, on all the factors, reported either improvement or much improvement. It should be noted, though, that some participants reported feeling a ‘bit worse’ with regard to their feelings about  themselves (one person) and their mood (one person). Where participants reported that things had not changed as a result of the football, it was in relation to sleep (four people) and family life (three people).  At a more global level, when the participants were asked to rate (on a ten-point scale) their physical and mental health overall, before and after joining the team, ratings suggested that their physical health had improved by over 56% (from an average rating of 4.56 to 7.11), and their mental health had improved by 100% (from 3.4 to 6.8).  Author interpretation and quotes in quotation marks: **Theme: Changes to mental health** Participants generally reported improvements to their mental health, one player commenting spontaneously on another player’s seemingly improved mental health. One particularly positive comment, from P3, was: ‘it’s helped me more than any medication could’. Other comments included: ‘I’ve felt I’m getting stronger both mentally and physically’(P3) and ‘It’s improved my ability to cope with the paranoia rationally, respectfully and maturely’. Yet comments appeared to be fairly realistic: ‘It’s obviously not the only thing but it’s helped’. (P2) **Theme: Self-confidence** Improved confidence and self-esteem were reported to be  benefits of the club by a number of players. In particular, one participant said: ‘It gave me the confidence to go for an interview, and then I got part-time work, now I’ve got full-time work. I don’t think I’d be in that position without the football […] I’m talking more in this evaluation than I would have before’. P4 also said ‘It gives you confidence’ and participants 1, 2, 3, 5, 6 and 10 all echoed this. P6 added: ‘my illness put me into my shell and football has improved that’. |
| Chowdhary, 2016 | None | None | None | The prevalence of depression was significantly lower in the Health Activities Programme (HAP) than Enhanced Usual Care. arm (adjusted risk ratio (RR) = 0.55, 95% CI 0.32–0.94, P= 0.01).  In the HAP arm as compared with the EUC arm, treatment remission based on the PHQ-9 scores was higher (for PHQ 55 adjusted RR = 1.63, 95% CI 0.91–2.99, P = 0.09) and the severity of depressive symptoms was lower (mean difference on the BDI-II of 6.5, P= 0.1), although these differences did not reach statistical significance.  (AI all) Briefly, regardless of their treatment status, most of the interviewed patients (n = 19/30) agreed that they found the treatment to be useful and that the counsellor helped them to address their problems. However, some patients who dropped out (n = 4/10) admitted that they had not understood what the treatment was about and were nervous about meeting the counsellor when asked to do so for the first time. // Our approach demonstrates four key points that may serve to guide future efforts. First, it is important to take account of local strategies and health beliefs that are relevant to the context of treatments, for example the fact most patients do not regard their symptoms as related to ‘mental disorder’. Second, treatment strategies derived from global evidence have cross-cultural applicability, for example behavioural activation emerged as a highly relevant theoretical framework for the treatment. Third, the delivery of treatment must take into account local structural and cultural barriers that might reduce engagement, for example lack of familiarity with ‘talking’ treatments and both opportunity and direct costs in accessing such treatments. Fourth, developing an acceptable and feasible psychological treatment depends on following a systematic process that includes reviewing the global and contextual evidence, consulting with patients, families, mental health experts and lay counsellors, and evaluating the intervention in clinical case series and pilot studies // |
| Abotsie, 2021 | None. | None. | 67% of participants (who returned questionnaires) reported making new friends | 67% of participants (who returned questionnaires) reported improved fitness, 58% reported reduced anxiety and stress and 42% reported improved mood.  Feelings:  Friendly: Provides people with a platform to build respectful, genuine relationships.  “My mental state is more positive due to this group, it gives me something to look forward to and gives me a sense of purpose”. (Participant 10, 29 years)  “I was met with a friendly bunch of lads and staff which has helped me with my communication skills and to be more relaxed about life”. (Participant 11, 43 years).  Feelings:  Inclusivity: Non-judgement and normalisation of their lives. Everyone is equal. There are no barriers.  “No judgment is passed on your capabilities or your mental well-being. I attend as many sessions as I can. It has also boosted my confidence and helped improve my people skills and socialising” (Participant 2, 24 years). |

**Table S6: Qualitative papers outcomes (n=13) Only outcomes included. Full qualitative tables available on request.**

| **STUDY ID:** | **Relevant social network data.** | **Other relevant data** |
| --- | --- | --- |
| Bradshaw et al, 1998 | Changes in social activity, confidence, and energy and interest in going out: Increase/ No Change/ Decrease Social Activity: 44%/ 56%/ 0% Confidence in Social Situations: 67%/ 33%/ 0% Energy and Interest in Going Out: 56%/ 44%/ 0% Overall Mean Change: 56%/ 44%/ 0% The most frequently ocurring benefits of seeing a volunteer stated by subjects can be broadly grouped under three headings: someone to talk to (n=5); help/support in going out (n=4); and increased awareness of personal strengths (n=3).  **Reasons given by subjects for how the befriending scheme had helped them** [split into mechanisms and outcomes - some both]**:** *‘Seeing the volunteer has made me pay more attention to my personal appearance.’ (1) ‘Having a guest coming to visit is nice.’ (1) ‘Seeing the volunteer has made me more aware of my own personal strengths.’(1) ‘I’m less anxious when I’m with my volunteer.’ (2) ‘I have thought more about things I used to do before I became ill.’(2)  ‘I feel more able to go out on my own.’ (2)* [...] more subjects reported increases in social confidence than those who reported increases in social activity, suggests that even those subjects who saw their befriender exclusively at home may have found the opportunity to talk to someone beneficial for their social confidence possibly from having the opportunity to practice their communication skills. (AI) *'I look forward to seeing the volunteer and having a good chat' (4) 'It's nice to have someone to talk to' (6) 'I like having company and someone to talk to' (7) 'I spend a lot of time planning where we can go together' (8)* | None |
| Fieldhouse, 2003 | The participants described the benefits of regular access to a social network characterised by friendships and reciprocal support, Norman: [Tim] might say ‘Oh I’m having trouble with my flat’ – and because I’ve been in the same boat – or might have been – I can at least empathise with what he’s going through. peer learning and modelling, and the freedom and confidence to improvise and experiment: Kevin: When you’re with other people you can try things out – can’t you, really. ‘Reality testing’ I believe it’s called ... I mean, you get some feedback to what you say, hopefully. You wouldn’t get it if you walked down the street and spoke to somebody because they’d think you were a lunatic or something.  AR: The participants’ descriptions of enhanced mood, reduced arousal and improved concentration which were directly linked to the environment. | **AR:** Rather than segregating clients and compounding society’s stigmatisation of them (Dunn 1999), services may be better equipped to integrate individuals with their wider community if they harness mainstream occupations. This study points to the benefits of using dedicated groups within ordinary settings to promote social inclusion and recovery for people with serious mental health problems   **Implementation: AR**: If clients’ pathways into these are navigated using the care programme approach (DH 1990), it enables statutory services to access a supportive community infrastructure (Hemming et al 1999) while retaining a high degree of flexibility and responsiveness in their delivery of care. Practitioners get to know clients well and the chances of early detection of distress and relapse are increased. Adaptive ways of asking for help are also reinforced because support is continuous rather than being activated by crises. |
| Felton et al, 2009 | 1. Observation: Photographs were often passive. Participants referred to seeing others doing activities, positioning themselves as an observer of others engaging in the social world. (AI) 4. Aspirations: Many photographs highlighted further hopes and aspirations for their future. However, money was seen as a barrier for realising this, as well as recognition that this would often mean doing things alone. (AI) The networks that the residents had access to were less likely to include people who had influence, power or access. This limits opportunities and could make people more vulnerable to prejudice and hostility in their communities. This might reflect the significance for participants of saying hello in local shops, which helped them to feel connected, secure and safe in the area. (AI - in discussion) | 3. Past: Narratives accompanying photographs often described past achievements, and involvement in activities. This was located historically rather than in the present. (AI) |
| Friedrich, 2018 | Participants make a wide range of connections through the scheme, both within it and, in some few cases, beyond it. A minority of participants found opportunities in employment and education as a result of attending the intervention: most who found exit routes did so through a wide range of other services. Participants highlighted a sense of feeling connected and valued as a result of participating and the relationships they developed. Critically, they felt connected to and part of a group with a ‘family atmosphere’ (P22) with benefits in the wider world. It motivated many to confront their isolation and contribute more widely both at the scheme in other areas of their lives. “Coping Through Football has in a way sort of changed me as well and ah I've become more like open ... make me more like feel better as a person and ah being a good friend to other people in Coping Through Football.” (P5) | **Emotional wellbeing:** “it's helping me a lot with my depression now. I feel it's not let me...I mean I know that trauma is never, it's never going to go. It just helps me with that...It helps my wellbeing in that I engage with more people .... When here, when doing this, I don't feel alone, I don't feel like I am a bad person, like I am the worst person in the world. Here I don't think anyone really judges me for that.” (P21). **Confidence/social skills:** Participants reported greater confidence and an increased belief in their own skills. “It's helped feel more confident in myself and, like, helped me to interact with people easier.” (P3). **Changes in lifestyle and physical health.** **New life skills**: Some participants mentioned having acquired additional life skills such as equipping them to support other people in emotional crisis, as well as plan and manage their time. |
| Hanlon, 2019 | AI: All patients with moderate or major improvement developed positive relationships with other people following their interaction with the CLP. This was described as a change from the isolation experienced prior to the intervention. These relationships were often formed through group activities which had been suggested or organized by the CLP. AI: Confidence: Some described gaining competence in new activities or rediscovering previous interests as a result of the Links Worker Programme. These patients usually also reported moderate or major improvement overall. These activities were often in organized groups, and the described impact was often a reflection of both increased social interaction and a sense of greater confi- dence in undertaking specific activities. Patients who rediscovered a sense of com- petence in an activity often described an increased sense of confidence and control over other areas of life. Some times this encouraged future action rather than actual action within the timescale of the study. | AI: Six described moderate or major improvement impacting daily life, two described slight improvement not affecting daily life and four described no overall change. Many of the difficulties experienced by patients stemmed from a lack of supportive relationships. |
| Hanly et al, 2020 | First, **hope and inspiration** describes how mentoring inspired a greater belief that recovery is possible. Witnessing their mentors’ successful recovery increased hope that they too could be freed from the cycle of chronic illness and relapse. One 19-year-old participant (Talia) commented that since meeting her mentor, she was no longer *“scared of [her] twenties”*, which she had previously assumed would be characterised by illness and isolation. Another participant, Michelle, stated: “I think it was just inspiring that no, actually this is a life that maybe I do want more than I want to be sick. **Motivation and agency** describes an increased readiness for action. Margie described: *“My brain is starting to recognise that healthy, happy, is actually worth something.”* Mentees reported that their involvement with mentoring motivated them to commit to recovery focused actions, included self-care activities, social eating challenges and reaching out to friends. Michelle offered: “It was nice to have someone to…go and do activities with and kind of give things a go and have a laugh along the way.” Nicole reported: “I got in touch with one of my really close friends who doesn’t live too far away and…[asked], you know, ‘If I could… if I need to talk to someone, could I talk to you?’ The next subtheme is **re-engaging with the world and others.** Mentees typically described their illness as profoundly isolating. Mentoring provided a bridge to support the transition back to a more connected and “normal” life. The mentoring sessions were an opportunity to socialise free of the imperative to pretend they were well, and a chance to increase tolerance for leaving the house and exploring being out and about. Jasmine said: *“I just have anxiety around crowded restaurants… and at the start we’d be like, let’s go somewhere else, but now I can just deal with it.* | The data in this study suggests these social benefits were afforded to those mentees who enjoyed a connected and harmonious mentoring relationship. The matching process attempted to ensure this outcome for all mentees, by offering them opportunity to select matching criteria of most importance to them. However, the restricted size and diversity of the mentor pool as well as (often unknown) pre-existing interpersonal patterns and preferences limited the assurance of achieving successful relationship pairings in all cases. Mentoring was consistently perceived by mentees as a more relaxed form of recovery support than clinical treatment. The elements of spontaneity and fun reported by mentees in the current evaluation was also found in Lippi’s [36] study. These elements may be particularly important in eating disorder treatment, due to the high level of monitoring and directiveness necessary in clinical treatment, which can be perceived as highly stressful and controlling [48] Research suggests that as relational vulnerability increases with personal disclosure, so too does the risk of job stress, burnout, and relapse [18, 19, 31]. Mentees in this study indicated that their mentors managed this balance successfully from their (mentee) perspective at least. In the words of Solomon [47], it could be said that these mentors were “expert at not being an expert”. However, mentors indicated this significant balancing act was not always achieved with ease, and they concurred that the training and support provided by EDV was critical to managing this challenge. The most commonly reported challenge of participation reported by both mentees and mentors was the sadness and loss due to separating from their mentor at program completion. Previous mentoring literature noted that there can be difficulties with ending the mentoring relationship due to the bond that often develops [51]. The difficulty associated with gaining then losing a significant source of support (for mentees) and a meaningful relationship (for mentors) should be carefully considered as a potential hindrance to balance against potential gains. |
| Hassan, 2020 | Social inclusion and connectedness  How participants framed the impact of the Life Rooms was wide ranging. However, what was discussed in depth was how the Life Rooms enhanced social inclusion and connectedness. The Life Rooms were described as social hubs for the majority of participants. Having library facilities, group meeting spaces and a café enhanced these social interactions further.  “I met someone there and we clicked and we had lunch in the little coffee shop there and it was like oh my god this is the first time in my adult life I have sat and had lunch with a friend” FGD6_SU.  Participants reported on how this social environment supported them feeling less isolated and lonely. They shared that the Life Rooms helped them in getting out of the house to a place where they are around others who have shared experiences. Some interactions happened instantly with other services users and, for some, these may have taken time to happen. Participants appreciated being in a place that allowed them to engage in activities on their own terms and respected their preference of wanting to be alone at times.  “When you’re going through a mental health issues, you feel so isolated – you are the only person that this has happened to – until you come to places like this and you think, ‘Oh… I’m not’… that feeling of isolation can sort of go then.” FGD2_SU.  “I have come here a few times just to be by myself, to a safe space which is nice because obviously I can come up and get a cup of coffee if I want and sit down and they respect the fact that you want to be on your own” FGD5_SU.  Relationship building is encouraged at the Life Rooms. Within courses and social groups, participants had time to interact with other individuals. They also highlighted that further interactions were encouraged by the Life Rooms staff through the introduction of service users to each other.  “Now like I said before coming here today has allowed me to meet six other people and talk a bit whereas before I would be sat at home trying to find a 9 letter word in Countdown” FGD1_SU.  Participants reported on the relationships that have been created through the Life Rooms and also indicated that relationships within their own families had improved. Since the Life Rooms is open to all, some participants reported how they had attended groups and courses with family members. Other participants reported on how the Life Rooms has given them the confidence to create new relationships. These interactions and relationships were key in enhancing participants becoming more involved and active.  “I went through a lot a hard time about 5 or 6 years ago and I lost a lot of friends because of it and since coming here I’ve made loads more friends” FGD4_SU. | Moving forward: self-development and independence  Another way in which participants described the Life Rooms impact was in the way it shaped their personal goals for the future. Participants describe a journey of self-development, which included self-awareness, learning to manage and look after oneself, gaining new skills, developing new hobbies and interests, enhancing confidence and gaining independence. Participants described change from not feeling the need to sit by the door when attending workshops, being able to get dressed and leave the house, and being able to complete day to day tasks independently.  “I am out of bed, I am dressed, depending on the day whether I’m able to cope with the shower but certainly washed, dressed and today I am out the house, I am here” FGD1_SU.  “I am able to go in and actually do my shopping instead of having to rely on people, getting my independence back is huge for me” FGD4_SU.  For many participants, self-development consisted of building skills and strategies, particularly in relation to mental health. This included developing confidence and self-esteem, or learning how to relax or manage their situation. Participants described how the different coping mechanisms that they have learnt and used gave them a sense of control and enabled them to deal with their situation.  “I would not have been able to do it if I hadn’t have gone to Life Rooms, it give me coping mechanisms, it’s given me strategies, and its helped me to get the confidence and self-esteem because I was at rock bottom” FGD1_SU.  Participants spoke about how the Life Rooms had facilitated a process of self-exploration and learning, which for many included being able to recognise their own strengths and weaknesses and being able to face life’s challenges. This was important for many participants because it allowed them to change their behaviour in order to manage their distress more effectively, for example through understanding when they needed help or being able to communicate their needs.  “I am now seeing things differently about myself. Since doing these courses I understand my illness more and I understand if I am having a bad day. It’s also helped me be able to voice things better as well. I can tell people more about my mental health….Even if it’s the middle of the night and I’m struggling, I know that there are people that I can phone and just say ‘I am not feeling great.’ So that’s how it’s helped” FGD4_SU.  Moving forward, participants described feeling more confident to work towards their personal goals, which included working towards employment, coming off benefits and pursuing further hobbies and interests. “I got asked if I wanted to run my own group because I spoke to a few of the people who work here about my experience… I would like to work again as you get that like self-esteem back” FGD3_SU. |
| Lund, 2019 | 2. A sense of belonging: mutual support and understanding.  Subcategories:  Sharing openly in a safe context  Good and bad days: group acceptance and understanding  **Expanding one’s social network**  Bonding and healing through humor.  **AI (unless quotes used)**  The experience of making new friends or deepened connections through the group was meaningful for participants. Group leaders could also encourage participants with tips and support for making contact with people outside the group. A feeling of community was valued and participants found ways to support each other, such as going on walks together, supporting each other with certain goals, and visiting a member who could no longer attend the group. Participants reported that getting to know people better at their care center was valued, especially when they struggled to make and maintain friendships on their own. Knowing they could meet at the care center could take the pressure off from needing to reach out on their own, and these relationships could develop into a friendship during or after the course as they continued to see one another. **Making friends who understood the need for flexibility around the good and bad days was valued. Nina said, ‘It’s good to meet friends from here, because you don’t need to be afraid to call up and say, if we had decided to … have coffee somewhere, that no, it doesn’t work today … [And they understand?] Yes, because they have been there too.’** | **3. Re-valuing Self: respect and self-worth.  Subs: Facing old views and prejudices; Feeling valued; respecting self and competencies; Purpose and self-worth through helping other. Facing old views and prejudices** Viewing mental illness from different perspectives, including personal recovery as a unique individual journey, was a topic in the course. This seemed to open a meaningful dialog in the group that helped participants to challenge prejudices they had experienced previously. Participants often reported that people in general tend to understand physical limitations, but if one has a mental disorder, there is less understanding. Examples given by participants included the feeling of being treated differently due to their diagnosis, or that employers, neighbors, friends, and/or family members did not understand their needs or limitations. Nina said that she felt a difference at work after telling her boss she had ADHD: ‘I’ve worked in the same place… for 16 years. And suddenly it’s like what I say doesn’t hold as much value as it did before.’ Group discussions could be meaningful to participants as they could start to de-identify with negative prejudices, and gain a more positive perspective of Self. Anita said that when the productivity topic was first presented, she thought, ‘I don’t do anything!’ The group helped her re-assess this old belief: 'When someone is on sick leave, it’s like aw, you’re just at home and don’t do anything. But you aren’t… But everyone thinks that and in the end you think that yourself… It can be a little disheartening, like you don’t do something worthwhile… So it was really nice to find out that I actually am really productive!' Sharing experiences with an understanding group supported the meaning-making process as participants challenged stigmatizing views that they had internalized, and had contributed to a lower sense of self-value. By challenging these negative views and focusing instead on their abilities, participants could develop a more positive view of Self. **Feeling valued: respecting self and competencies** Past negative experiences had affected participants’ self-confidence, and seemed to contribute to self-devaluation. It was therefore meaningful to feel heard and respected by the group members and group leaders, especially when they felt they had not been listened to, or believed, by health care personnel or others in the past. Being appreciated by health care workers and peers for one’s strengths was valued, as was being treated ‘as equals.’ It was common to take one’s natural talents for granted while focusing instead on what one could not do, or struggled to do. Participants often found it easier to accept others’ faults and identify others’ positive attributes than their own, but group participation allowed them to pay witness to fellow members’ journeys, and to start to identify their own strengths and competencies. Hanna’s struggle was evident when she compared herself positively to another group member: ‘Maybe it’s horrible to say… sometimes I felt that, yeah, this is something I’m good at, which is something the other person isn’t good at…maybe it’s dumb to feel…that this is something I can do at least.’ Feeling valued and respected by the other group members for being ‘good at something’ or ‘clever’ seemed to help participants acknowledge their strengths and value and respect themselves, which added to a sense of meaning.  **Purpose and self-worth through helping others** Participants often reported that after many years of struggles with mental health, their self-esteem and perceptions of self-worth suffered. Many described feeling ‘useless’ or ‘worthless’ in relation to having a disability. However, when they could give ‘useful’ advice to others in the group, participants realized their experiences could help others. It was then possible to gain a new perspective on difficult life experiences as valuable. This related to mutual support described under Belonging, but brought in the component of re-valuing as people reflected on their feelings of self-worth. Being able to help other group members was meaningful and contributed to a renewed sense of purpose. This could foster a more positive view of Self and re-frame life experience as valuable lessons. Ben reflected about the group: 'It’s like hard to explain… [It] has to do with self-confidence. I think that it applied to most of [the others too], that people listen to what you say, and you notice, and everyone wants to be good, and it just works. Self-confidence really grew, both by listening and talking, it really did, strange. …I was 20 when I had my first psychosis… it’s been 35 years, and many years in the hospital, group homes, and a lot. So I haven’t experienced something this positive, ever. And it really depends on the personnel who were part of the group, they listened to us/ …/But what means most for the future… is the kick I got before talking with people, in a good way, a useful way… Could give some advice that was beneficial for others, and same for them, they also taught from their experiences.' In conclusion, re-valuing self built upon joining and belonging. By processing old experiences and prejudices, and being valued as a person with competencies,  participants could re-value their strengths. Participants reported that by being able to help others in the group, they experienced improved self-worth as well as were able to re-frame previous life experiences as useful. Helping others seemed to create meaning through experiencing a sense of purpose. |
| Mathias, 2019 | I - Young people commonly described the formation of new peer friendships. Young women said that after meeting others, they would visit each other’s houses to study together, as a young woman describes below: (E) Now we have been meeting up and walking to school together. And actually, we even held a small party for a group of us a while ago, which we didn’t tell our parents about. We played a game and we were all laughing like crazy. // Other examples of the benefit of friendship networks included a group of girls who jointly enrolled for distance-learning style schooling together and four young men who had agreed jointly to stop using drugs and alcohol, reporting that peer encouragement helped them to stay ‘clean’ // The young people reported increased participation in community activities and events because they were treated with respect, and this gave them the courage to participate in other public events. Secondly, the Nae Disha module proposing collective action for community change helped them to recognise that young people could be legitimate agents of social change. A young man described how the group provided the opportunity to work collectively, as quoted below: (E) As we all live near to each other we have continued to meet up regularly outside of the group meetings. But the group gave us opportunities to make decisions together, like members of a government collaborate to bring change. // I - Young men described how participation in the group had improved community members’ perception of them. Young men who had been called ‘nashedi’ (addicts) told us that they were now called ‘sewadhar’ (students). They also said that since joining the group and reducing substance abuse, they were permitted to enter the gurudwara (Sikh temple), whereas previously they were not permitted to enter. Conversely, several young men said that attending the group resulted in some negative judgements by people in the community, as the group was considered to be for mad people, or people with poor character. This is described by one young man below: (E) One day one of my friends also came with me to the group. The next day he told me that when he went back (home) after the group, people teased him and called him names. They said that the boys in the group were not considered to be good boys and due to this problem, he couldn’t continue in the group // | I - Participants and parents described how the young people demonstrated greater self-efficacy after participating in the groups. For example, several young men said that they had developed the confidence to apply for new employment options // I - The young people described how their mental health had improved because of the intervention. As well as reporting less anxiety and sadness, and fewer somatic complaints, young people also said that they were better able to manage their frustration // I - Peer facilitators and young women described how the young women became more confident and consequently could move around more freely. This included embarking on new journeys, such as a school girl in Mussoorie who negotiated with her father to be allowed to walk to the library, so she could attend a computer course there. Others reported that they had started walking alone, as described below: (E) Earlier, to go to school my mother would walk with me and also she would have to come to pick me up, but after joining the group I told her there was no need, and now I go by myself // I - Female participants described that they grew in confidence to speak publicly and participate in family decision-making. |
| O'Brien, 2011 | The participants also outlined the personal social benefits derived from meeting other people, joining in the activities and working as part of a team. The most highly treasured outcomes were direct involvement in the community, taking responsibility for the environment and developing respect for it.  The Meanwhile participants seemed to wish to engage in building social capital within the group. They also gradually developed bridging links with their diverse community and cultivated a self- directed effort towards becoming stewards for the environment and agents of change. This aided recovery from ill health, rehabilitation in the context of personal health and inclusion into the local community. However, they also encompassed ‘ecological understanding’, ‘green space conservation’, ‘environmental literacy’, ‘community involvement’ in the context of public health and environmental recovery and sustainability. as one participant stated: ‘... yes it’s a direct help that I’m doing and I’m participating in the community. I am conserving all these living things... I can say, without going into detail, that there’s a lot of benefits in what I am doing: for nature and the community in general.’ (Homeless participant) Outcomes from the targeted intervention were social capital and inclusion and greater community acceptance for a sector of society that is often marginalized: ‘I am part of a group, a city, a country and a society which can take care of its vulnerable members, of which I was one, and this has benefited me greatly and I feel I want to give something back to this culture and to nature in general by coming here and making an effort.’ (Volunteer recovered from mental health problems) | Participants described deriving personal health benefits but also talked about valuing their newfound or existing relationship with nature. The mutual nurturing that they gained from this amplified their wish to develop the relationship into stewardship of the environment, encouraging wider community involvement in respecting nature and taking care of the environment. On a personal level participants found their relationship with nature to be therapeutic, rewarding, facilitating spiritual growth, allowing them to develop a sense of pride and helping them develop a sense of self and sense of place. This hands-on contact with nature helped them to accept and better cope with their illnesses/difficulties and develop some resilience.   A trainee at Meanwhile found respite from depression and was helped to make progress from being housebound: ‘It helped me get out the house and meet people and join in the activities a bit more. I don’t know if you’re aware, I had depression, I wouldn’t go out at all, I mean it’s about a year ago, wouldn’t go out at all...’ (Mental health participant) Skills development, potentially leading to employment, were valued by participants at Meanwhile as they gained new knowledge which improved their self-esteem and provided greater opportunities for employment, as identified by a mental health participant: ‘I hope to get a bit more training this year... formal, not just on-the-job training... I would like to do that. I think I’d like to do this as a job, rather than drive lorries, too much stress... I don’t want to be stressed anymore.’ (Mental health participant) |
| Sheridan, 2018 | I - For some, it inspired action to enhance connectedness and expand social networks: (E) I do not think I have changed in any way as I had no person to join up with. However, towards the end of the study I have met up with a few girls from [a club] and feel I may be able to meet with them. (SOG, female, age 40s) // I - VPG participants identified programme activities as things they could also do with other friends or family members, suggesting the increased capacity to socialise attained from the programme was a transferable skill that could be exercised after the study had completed: (E) I could bring the children to some of those places … I know Dublin very well now. (VPG, female, age 40s) // I - Participating, for both groups, built confidence in socialising, which ultimately resulted in people feeling more comfortable in social situations (e.g. speaking to people in cafes, shops and on public transport): (E) I learned to interact and communicate with people. It made me feel more confident when socialising. (SOG, female, age 40s) // I - For the VPG, tangible benefits were as follows: (1) having something to look forward to, (2) feeling challenged and succeeding, (3) experiencing life outside of the home and (4) relating to others during activities. Simply having the financial means and motivation to connect with other people enabled SOG participants to set goals, develop relationships and engage with their communities. The programme appeared to open doors for participants into a social world previously inaccessible to them // I - There was evidence that achieving a degree of confidence and feeling comfortable in varied social situations, as a consequence of participation, translated to other social contexts. Taking part in the programme assisted people to engage with their community, plan future activities and develop social aspirations beyond the study period. | I - Demonstrating the capability to socialise for both groups buffered against societal beliefs participants perceived relating to their absence of social value, unlikely recovery and inability to manage the responsibility of balanced friendship. // |
| Suto, 2020 | Community gardening offered possibilities for current and future engagement with other people, as Ernie explained.  You meet people, you have similar interests, and things are kind of interconnected in a community, right? You can of course connect with people, everyone has pretty much a wide range of interests; you might find other things that you’re interested in. That’s part of the networking, socializing thing.  Some people gardened as a means to socialize; thus the occupation was less important than the opportunity to interact with people. Yet for others who experienced anxiety, their efforts were more on the garden activities and less on social interactions. Hannah said, “Yeah, we just sort of make chitchat. Some people just talk about whatever is going on in their life. Yeah. Mostly I just kind of stay focused on plants.” Thus, positive feelings appear linked to having choices, to use the occupation as an end or a means.  Creating a Sense of Belonging:  The experience of belonging that participants perceived arose through inclusion and affirmation from others, mutual support, social interaction, and connection to others through interpersonal relationships. Role development occurred formally around assigned gardening session tasks and chosen activities (e.g., making tea, getting supplies out, and mixing soil). Roles evolved informally and participants spoke proudly of them. Joel said:  “I have another role in the garden. I’m the unofficial photographer.”  Fulfilling roles contributed to an atmosphere of belonging, which further strengthened an individual’s sense of purpose and place within the group through validation from others. Fred observed:  People like to call me the caretaker. I’m the guy that goes out and waters and that’s why we had such a good garden. Watered every day, right . . .and it feels like people are counting on me too. I don’t get that very often because I don’t make commitments. I don’t really pay that much attention to people here because I’m a recluse.  Stacie noted that participation in the garden project fostered a sense of belonging and being needed: “It was nice to know people were expecting me to be there on time. So, I had something to do at least once a week, to um, prepare for class, and to be reliable.” Some participants’ circumstances prevented them from regular attendance, but they stated their intention to resume gardening. Such expressions of commitment reinforced participants’ experiences of feeling included in something larger than themselves (i.e., the community garden) and emphasized their belonging to it. Mutual support occurred when people watered for others and harvested their produce. Matthew explained:  Well, yeah, because it works both ways for people. Um, if you help, if you learn how to help people and how they can help you, cause it’s a two-way street. And it’s good because you know each other and at the end of the day, you feel good. The community garden provided opportunities for participants to share produce with others and to engage in social interactions where the occupation was the explicit focus rather than the socializing. Thus, participants created relationships through their actions in a place where they were comfortable and felt they belonged. Brian summed up this phenomenon:  I can garden and talk to people whereas there’s not a lot of things that I can do where I’m doing both. If we’re gardening in one plot and there’s two or three of us then, you know, I can do the gardening and be talking to them as well and it just seems to flow really well. | Cultivating Positive Feelings Through Doing:  This finding highlights the benefits that individuals attributed to their participation in the community gardening program; in short, they recognized the positive outcome of “doing.” The perceived benefits included participants feeling more confident about their abilities, trusting their capacity to learn, and feeling better about themselves. Carmalea stated:  There were times that I’m really depressed you know and every time I go to the garden, because I see my plants growing, that makes me feel good. Because that reminds me that I did something good at least. You know, at least, this one I didn’t screw up.  The participants reported mainly positive feelings specifically joy, happiness, and pride. The data identified doing as a means of eliciting pleasant feelings. Tanya explained:  I feel really happy when I garden. I love gardening. It makes you feel like you’re accomplishing something. It makes you feel happy because when you see your plant growing and doing well [you recognize] that you helped make all that happen.  Relaxation and stress reduction were frequent outcomes of doing community gardening in the context of this program and its welcoming milieu. Participants also identified irritations associated with gardening, yet remained optimistic. As Carl  stated: “There’s all kinds of negative feelings that go with any project . . . you know, nothing big; the beneficial feelings outweigh the negative. The negatives are small little things, like swatting at a bug.” Recognizing the frustrations that Carl raised, Brian highlighted the relaxing features of gardening.  It’s pretty low-key. It’s pretty calm, like you just go there and you get your own plot. You can’t really screw it up. I mean you’re just planting seeds in mud. . . . I just think gardening is calming. I don’t know, a lot of people don’t spend much time outside and it’s just healthy to get outside. To get some fresh air. Get some sun. |
| Tarbet, 1985 | PreGroup SN Size: 20% = 7; 50% = 6*; 10% = 5; 10% = 3; 10% = 0; *Excludes one participant's extensive network of 'good friends' through AA and MH programs and another participant's network of acquaintances through work and 2 groups. [no clear measure of postgroup SN size but] All members interviewed added from approximately 1-10 locally accessible friends to their social networks [...] All but one of these members said they developed at least one friend through SYP [though not all would apply the term 'friend' to those they know from group]. John had '*dramatically more contact'* and that part of his decision '*to get help involved being in more contact with friends. The group has provided a meeting place, a structure to enhance relationships, a place to meet new friends; its made me more aware of the friends I had; it gives a structure to work with other people; and it promotes mental health, therefore increasing my contact with friends.' I have a lot more contact [with a wide range of friends] than I did before joining the group* *(Laura)* Most of the new friends were made through the group or through other activities related in some way to the mental health system. These clusters were often dense within themselves but had few exchanges or interactions with family clusters. All members interviewed reported increased interactions with friends since joining the group. Interactional changes with family members were mixed [...] 5 members did report close contacts with family since joining the group, 2 reported decreased contact and 2 reported no change [phone and face-to-face]. The interviews strongly suggest that postgroup networks provided members with a greater sense of intimacy than previous networks: All members made friends they felt could understand them; 4 members reported an increased satisfaction with the amount of interaction with friends and kin, though 3 still had less than preferred. 7 members postgroup felt part of a close group of friends (only 1 did prior to joining) and 3 did not feel this way pre or postgroup. As a group, members would with much greater frequency than before turn to friends for their first source of help, suggesting a decreased intimacy [or dependence] with family and an increased sense of intimacy with friends. Every member interviewed said that after participation in the group, he or she had new friends who understood his or her experiences with mental illness and psychiatric services. | Janet said after one friend had seen the value of self-help for Janet, she had begun to think about joining a support group for herself. *I can handle more than I think I can (Dorothy).* |

**Table S7: Quality appraisal – qualitative studies (n=13).**

| **STUDY ID (Author last name, year)** | 1.1. Is the qualitative approach appropriate to answer the research question? | 1.2. Are the qualitative data collection methods adequate to address the research question? | 1.3. Are the findings adequately derived from the data? | 1.4. Is the interpretation of results sufficiently substantiated by data? | 1.5. Is there coherence between qualitative data sources, collection, analysis and interpretation? |
| --- | --- | --- | --- | --- | --- |
| Bradshaw, 1998 | Yes | No | Yes | Yes | No |
| Felton, 2009 | Yes | Yes | Yes | Yes | Yes |
| Fieldhouse, 2003 | Yes | Yes | Yes | Yes | Yes |
| Friedrich, 2018 | Yes | Yes | Yes | Yes | Yes |
| Hanlon, 2019 | Yes | Yes | Yes | Yes | Yes |
| Hanly, 2020 | Yes | Yes | Yes | Yes | Yes |
| Hassan, 2020 | Yes | Yes | Yes | Yes | Yes |
| Lund, 2019 | Yes | Yes | Yes | Yes | Yes |
| Mathias, 2019 | Yes | Yes | Yes | Yes | Yes |
| O'Brien, 2011 | Yes | Yes | Yes | Yes | Can't tell |
| Sheridan, 2018 | Yes | Yes | Yes | Yes | Yes |
| Suto, 2021 | Yes | Yes | Yes | Yes | Yes |
| Tarbet, 1985 | Yes | No | Can't tell | Yes | Can't tell |

**Table S8: Quality appraisal – Randomised controlled trials (n=17)**

| **STUDY ID (Author last name, year)** | 2.1. Is randomization appropriately performed? | 2.2. Are the groups comparable at baseline? | 2.3. Are there complete outcome data? | 2.4. Are outcome assessors blinded to the intervention provided? | 2.5 Did the participants adhere to the assigned intervention? |
| --- | --- | --- | --- | --- | --- |
| Ammerman, 2013 | Yes | Yes | Yes | Can't tell | Can't tell |
| Bitter, 2017 | Yes | No | Yes | No | Can't tell |
| Calsyn, 1998 | Can't tell | Can't tell | Can't tell | Can't tell | Can't tell |
| Castelein, 2008 | Yes | Yes | Yes | No | Yes |
| Chang, 2016 | Yes | Can't tell | Yes | Yes | Yes |
| Fowler, 2018 | Yes | Yes | Yes | Yes | Yes |
| Garety, 2006 | Yes | Yes | Yes | No | Can't tell |
| Gater, 2010 | Yes | Yes | Yes | Yes | Can't tell |
| Haslam, 2019 | Yes | Yes | Can't tell | Can't tell | Yes |
| Johnson, 2018 | Yes | Yes | Yes | Yes | Yes |
| O’Connell, 2020 | Can't tell | No | No | Can't tell | Can't tell |
| Rivera, 2007 | Can't tell | Yes | Yes | Can't tell | Can't tell |
| Sheridan, 2015 | Yes | Yes | No | Yes | Can't tell |
| Tempier, 2012 | Yes | Yes | Can't tell | Yes | Can't tell |
| Terzian, 2013 | Yes | Yes | Yes | No | Can't tell |
| Thorup, 2006 | Can't tell | Yes | No | Can't tell | Can't tell |
| Varga, 2018 | Can't tell | Yes | Can't tell | Can't tell | Can't tell |

**Table S9: Quality appraisal – other quantitative (n=12)**

| **Non-randomised quantitative studies** | | | | | | | | | |
| --- | --- | --- | --- | --- | --- | --- | --- | --- | --- |
|  | 3.1. Are the participants representative of the target population? | | 3.2. Are measurements appropriate? | | 3.3. Are there complete outcome data? | 3.4. Are the confounders accounted for in the design and analysis? | | 3.5. During the study period, is the intervention administered (as intended? | |
| Mazzi et al, 2018 | Yes | | Yes | | Yes | Can't tell | | Can't tell | |
| Becker, 1998 | Can’t tell | | Yes | | Yes | Can’t tell | | Can’t tell | |
| Hacking, 2008 | Can't tell | | Yes | | Yes | Can't tell | | Can't tell | |
| Bailey et al 2020 | Yes | | Yes | | Yes | Can't tell | | Yes | |
| Petryshen (2001) | Yes | | Yes | | Yes | Can't tell | | Can't tell | |
| Webber, 2019 | Yes | | Yes | | No | Yes | | Can't tell | |
| Haslam et al., 2016 | Can't tell | | Yes | | No | Can't tell | | Can't tell | |
| Bjorkman, 2000 | Can't tell | | Yes | | Can't tell | Yes | | Yes | |
| Fitzgerald, 2011 | Yes | | Yes | | Yes | Can't tell | | Yes | |
| Aggar et al 2021 | Can't tell | | Yes | | No | Can't tell | | Yes | |
| Webber et al 2021 | No | | Yes | | Yes | Can't tell | | No | |
| **Descriptive quantitative studies** | | | | | | | | | |
|  | 4.1. Is the sampling strategy relevant to address the research question? | 4.2. Is the sample representative of the target population? | | 4.3. Are the measurements appropriate? | | | 4.4. Is the risk of nonresponse bias low? | | 4.5. Is the statistical analysis appropriate? |
| Segal & Holschuh, 1991 | Yes | Yes | | Yes | | | Can't tell | | Yes |

**Table S10 Quality Appraisal – mixed methods studies (n=12)**

|  | 5.1. Is there an adequate rationale for using a mixed methods design to address the research question? | 5.2. Are the different components of the study effectively integrated to answer the research question? | 5.3. Are the outputs of the integration of qualitative and quantitative components adequately interpreted? | 5.4. Are divergences and inconsistencies between quantitative and qualitative results adequately addressed? | 5.5. Do the different components of the study adhere to the quality criteria of each tradition of the methods involved? |
| --- | --- | --- | --- | --- | --- |
| Bertotti et al, 2018 | Yes | Yes | Yes | Can’t tell | Can’t tell |
| Howarth et al, 2018 | Yes | Yes | Yes | Can't tell | Can't tell |
| Bragg, 2014 | Yes | No | No | Can't tell | No |
| Snethen et al, 2012 | Can't tell | No | No | Can't tell | No |
| Sexton, 1992 | No | Yes | No | Can't tell | No |
| Mak, 2016 | Yes | Yes | Can't tell | Yes | Can't tell |
| Kaltman, 2016 | Can't tell | Can't tell | Yes | Yes | Yes |
| van der Venter, 2014 | Yes | Yes | Yes | Yes | Yes |
| Margrove et al 2013 | Yes | Yes | No | Yes | Yes |
| Darongkamas (2011) | Can't tell | Can't tell | Can't tell | Can't tell | Can't tell |
| Chowdhary, 2016 | Can't tell | Can't tell | Can't tell | Yes | No |
| Abotsie et al, 2021 | Yes | No | No | Can't tell | No |
